# Effectiveness of dietary modifications in reversing damage induced by high-fat diet in rats

**DOI:** 10.1007/s13105-026-01205-y

**Published:** 2026-07-14

**Authors:** Manuel Jiménez-García, Maria Magdalena Quetglas-Llabrés, Susana Esteban-Valdés, Maria del Mar Ribas-Taberner, Antoni Sureda Gomila, David Moranta Mesquida, Silvia Tejada-Gavela

**Affiliations:** 1 Laboratory of Neurophysiology, Department of Biology, University of the Balearic Islands, 07122 Palma , Balearic Islands Spain; 2 Health Research Institute of the Balearic Islands (IdISBa), Palma, 07120 Spain; 3 Research Group On Community Nutrition and Oxidative Stress, University of the Balearic Islands, 07122 Palma, Balearic Islands Spain; 4 CIBEROBN (Physiopathology of Obesity and Nutrition), 07122 Palma, Balearic Islands Spain

**Keywords:** Antioxidant-rich diet, Fatty acid metabolism, Fatty liver, High-fat diet, Inflammation

## Abstract

Excessive dietary fat intake is a major health concern, contributing to the increasing prevalence of Metabolic Dysfunction Associated with Fatty Liver Disease (MAFLD) worldwide; different prevalence between sexes has been postulated but it is not clear. This study evaluated the effects of a high-fat diet (HFD) in Wistar rats and the impact of a transition to standard or antioxidant-rich diets in the same animals, exploring differences between sexes. Female and male animals were maintained for 5 months: control (chow diet) and HFD groups throughout the study, and two intervention groups switched after 3 months from HFD to standard or antioxidant-rich diets. Body weight was monitored, hepatic lipid accumulation, plasma levels of glucose, advanced glycation end-products, interleukin-6, and polyphenolic content, and hepatic oxidative stress markers (catalase, superoxide dismutase, glutathione peroxidase, and malondialdehyde) were measured, along with gene expression related to inflammation and lipid metabolism. HFD significantly increased body weight (females: 123.9 ± 13.57 g; males: 163.1 ± 9.97 g) and hepatic lipid accumulation (female proportion: 9.4 ± 0.4; male proportion: 10.5 ± 0.2) compared with control group (females: 28.96 ± 3.95 g; males: 86.29 ± 3.79 g, p < 0.01; 0% lipid deposits for both sexes), with higher effect on body weight in females. Dietary intervention led to a significant reduction in both parameters and for both sexes. Biomarkers of oxidative stress, inflammation and gene expression analysis showed that dietary modification reversed HFD-induced adverse effects in these parameters, restoring levels comparable to controls; with more consistent effects in animals of both sexes that switched to an antioxidant-rich diet. Indeed, this antioxidant-rich diet group showed higher levels of plasma polyphenols (females: 6.28 ± 0.46 ng/L; males: 6.85 ± 0.49 ng/L, p < 0.001) and a better antioxidant and inflammatory gene expression profile even compared with the group that switched to a chow diet. Overall, dietary modification mitigates several adverse effects of HFD, with antioxidant-rich diets showing greater efficacy across multiple endpoints, with similar effects in both sexes.

**Supplementary Information:**

The online version contains supplementary material available at 10.1007/s13105-026-01205-y.

## Introduction

The consumption of ultra-processed products and fast food are increasingly dominating worldwide, while the time dedicated to cooking healthier food is decreasing [1], leading to a potential global malnutrition [2]. Over the past decade, there has been an increase in studies on the effects that a high-fat diet (HFD) can have on the liver. In fact, foods with inflammatory properties, such as red meat rich in saturated fatty acids, and sugary drinks, have a high correlation with the development and progression of hepatic disorders [3]. There is significant concern about Metabolic Dysfunction-Associated Fatty Liver Disease (MAFLD), a term coined in 2020 to better define non-alcoholic fatty liver disease (NAFLD) and encompass its heterogeneity [4]. The current global prevalence of MAFLD is estimated at 32–39%, but these percentages are expected to increase in the next decades [5]. Although different prevalence between sexes has been described [6–8], sex-related risk differences is not completely clear [9]. This syndrome is characterized by excessive fat accumulation (> 5%) in the hepatocytes, leading to hepatic steatosis [10]. The excess triglycerides form lipid droplets inside hepatocytes causing cellular organelles to be displaced to the cell edges [11]. Along with fat accumulation, changes in the structure (i.e.: hepatocyte ballooning) and function of hepatocytes are observed, generating chronic inflammation and cellular damage [12]. Indeed, lipid accumulation in the liver promotes different processes such as mitochondrial dysfunction and endoplasmic reticulum stress, as well as the synthesis and release of pro-inflammatory cytokines, including tumour necrosis factor (TNF-α) and interleukin-6 (IL-6). These cytokines act as chemical messengers and can interfere with processes such as insulin signalling [12]. This type of interference may be due to the activation of inflammatory signalling pathways, the inhibition of insulin receptor tyrosine kinase, or altered translocation of glucose transporter type 4, promoting insulin resistance in the liver [12, 13].

Excessive production of reactive oxygen species (ROS) can occur in MAFLD, contributing to chronic inflammation. When ROS production exceeds the antioxidant capacity of the cells, it results in oxidative damage and cellular dysfunction. This situation promotes molecular oxidative modifications that result in hepatocyte injury and the activation of pro-inflammatory cytokines, ultimately contributing to liver fibrosis [14, 15]. Elevated levels of MDA, a marker of oxidative damage and a product of lipid peroxidation, in the liver and serum of patients with MAFLD correlate with disease severity, reflecting the degree of oxidative stress and liver damage. In addition, MDA can interact with proteins, DNA and other biological molecules, causing increased cellular damage, inflammation and liver fibrosis [14]. To counteract the excess of ROS, the body has different defences, such as several antioxidant enzymes like superoxide dismutase (SOD), catalase (CAT), and glutathione peroxidase (GPx).

Up to now, there has been a recognized lack in the pharmacological treatment of MAFLD [16]. Nevertheless, the Food and Drug Administration (FDA) has recently authorised Rezdiffra (resmetirom), representing an important advance in addressing this need [17]. Despite this pharmacological improvement, efforts are still focused on reducing different factors that contribute to the accumulation of fat in the liver, such as a hypercaloric or a fat-enriched diet, as well as a sedentary lifestyle. Nutritionally, a diet with anti-inflammatory properties and a reduced intake of refined or high-glycaemic carbohydrates can stabilise triglyceride and glucose levels, which in turn may increase serum HDL levels. This approach can improve insulin sensitivity, thereby lowering the risk of insulin resistance, and contributing to weight reduction and decreased lipid accumulation. Overall, it may ultimately mitigate liver damage both in the steatosis phase and in early cases of fibrosis [18, 19]. Some results in humans have pointed out that a Mediterranean diet, with high amounts of antioxidant components such as polyphenols, could help these patients [20]; however, clinical trials have shown inconclusive outcomes [13, 21]. Additionally, some studies have pointed out differences between females and males in several parameters evaluated in MAFLD models, such as in inflammatory cytokines and redox homeostasis [22], and liver lipid deposition [23]. Studies in animals focus on different comparative groups or on the administration of compounds [24–26]. Supplementation with active compounds has been described in vivo; for instance, polyphenol-rich extracts or resveratrol have ameliorated fibrosis and inflammation in mouse models of non-alcoholic steatohepatitis [25, 26], but no significant or conclusive effects have been observed in patients [21]. Therefore, investigating changes in the overall diet may be essential to better reflect real-life human conditions. Thus, the aim of the present work was to study the effect of dietary changes in a HFD rat model by evaluating the inflammatory and oxidative status of the animals, assessing the difference between sexes.

## Materials and methods

### Animals and treatments

Fifty-six Wistar rats (twenty-eight of each sex) 5 months old were housed individually in standard cages and maintained under controlled conditions (20 ± 2 °C; 70% humidity, and a 12-h light/dark cycle, with lights on at 08:00). All efforts were realized to reduce the number of used animals and their suffering. All procedures were carried out in accordance with the EU Directive 2010/63/EU of the European Parliament and of the Council, following the Spanish Royal Decree 53/2013 for the Protection of Vertebrate Animals used for Experimental and other Scientific Purposes, and the guidelines of the Bioethical Committee of the University of the Balearic Islands (approval reference number 2023/02/AEXP).

### Experimental design

**Fig. 1:**
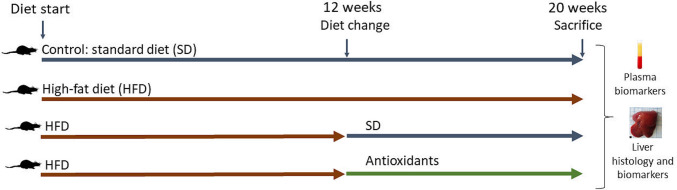
Timeline of the experimental procedure. Control rats were fed a standard food; one group of rats were fed a HFD for 20 weeks; the other two groups were fed a HFD for 12 weeks, and afterwards diet was changed to standard food or diet rich in antioxidants. The nutritional intervention was performed for both sexes. At the end of the experiment (five months), rats were sacrificed, and plasma and liver were used for histological and biomarkers determinations

The animals were randomly divided into four groups (n = 7 per group and sex). Treatments were based on different diets (Fig. 1). The control group was fed standard food (Panlab A04, Panlab S.L.U., Barcelona, Spain) which guarantee a balanced nutrition of the animals throughout the experiment (20 weeks). The other three groups were fed a HFD during the first three months since it has been described that is a lapse of time enough to induce non-alcoholic fatty liver in rodents [27]. During the next eight weeks of the experiment, the control group continued the same diet, one of the groups continued with the HFD, one changed to a standard diet like the control group, and the last group changed to an antioxidant-enriched diet (the chosen time was previously described to induce physiological changes) [28]. The HFD consisted of 25–30 g of high-fat food daily, including biscuits, red meats, cheese, or sweets [29]. The antioxidant-rich diet consisted of 25–30 g of fruits and vegetables (broccoli, apple, banana, blueberries) daily during the last eight weeks, to increase the intake of polyphenolic compounds [30]. The estimated nutritional composition is included in the Table 1. All groups had tap water and standard food (SAFE A40, SAFE Inc., Augy, France) ad libitum throughout the experiment. The animals were weight three-four days per week during the experiment.

**Table 1:** Approximate calculation of macronutrient and energy composition per day of the experimental diets

Component	Standard diet (SD)*	High-fat diet (HFD)	Antioxidant-rich diet
Energy (kcal)	60.05	85.96	14.85
Total protein (g)	2.7	1.572	0.391
Total fat (g)	0.576	4.314	0.086
Polyunsaturated fatty acids (g)	0.274	0.420	0.032
Monounsaturated fatty acids (g)	0.720	1.544	0.015
Saturated fatty acids (g)	0.088	2.176	0.030
Cholesterol (mg)	0	11.318	0
Fiber (g)	0.738	0.333	0.917
Carbohydrates (g)	1.8	10.136	3.055
Water (g)	2.178	29.538	24.51

* SAFE A40 (SAFE Inc., Augy, France). The data represent the total intake of each diet per animal

At the end of the experiment (20 weeks), the animals were sacrificed by decapitation to not to interfere with subsequent analyses. A sample of the liver was used for histology assessment. Plasma was collected in ethylenediamine tetraacetic acid (EDTA) glass containers. The rest of the liver and plasma samples were immediately frozen in liquid nitrogen and stored at −80ºC until analysis.

### Histology

Liver samples were fixed in 10% paraformaldehyde during 24 h for histological analysis [31]. The samples were placed in a 20% sucrose solution the previous day, to prevent tissue breakage. Samples were rinsed with PBS and embedded in a freezing tissue medium (LEICA, Ref: 14,020,108,926) on a glass plate. The Sects. (10 μm) were obtained through the cryostat (Leica CM1520), and they were placed in PBS to prevent damage. Sections were mounted on slides for staining. They were fixed and processed for oil red staining (10% formalin, 60% isopropanol, oil red solution, 60% isopropanol, distilled water, Mayer's haematoxylin, distilled water, and tap water). The red oil solution binds to lipid deposits, producing red spots [32]. Hepatic lipid droplets were quantified in a systematic manner to guarantee representative and reproducible results. In particular, four independent animals were analysed per group, and four distinct liver sections were evaluated from each animal. Within every section, five randomly chosen fields were used for image acquisition and subsequent analysis. Microscopy images were captured using a LEICA MZ16 equipped with a 10 ×/0.25 objective. Quantitative analysis of the images was performed with ImageJ, measuring the proportion of the red stained area. Representative images were selected from randomly chosen fields with adequate technical quality. Fields containing staining or sectioning artefacts were excluded. The selected images were considered representative of the overall observations as staining patterns were highly consistent within each experimental group.

### Plasma analyses

The determination of glucose concentrations in plasma was carried out following the kit manufacturers’ instructions (SPINREACT, S.A.U., Girona, Spain). The kit evaluates the increase in nicotinamide adenine dinucleotide (NADH), which is proportional to the glucose levels in the sample. Two reagents were used: R1, a buffer, and R2, the enzymes, along with a glucose calibrator. The content of R2 was dissolved in the bottle of R1, and the appropriate amounts of the working reagent were added to a microplate, along with the samples or the standard. The reading was performed at 340 nm in the spectrophotometer (Biotek, Epoch), and the glucose concentration was subsequently calculated using the following equation: (((A)sample – (A)blank)/((A)standard – (A)blank)) × 100 = mg/dL of glucose in sample, in which A is absorbance.

In order to study the concentration of polyphenols, the Folin–Ciocalteau method was used in plasma samples. When polyphenols react with this reagent, a blue coloration is produced, which is easily measurable. Acetone was used to deproteinise the sample. Then, samples or standards were added to a solution of distilled water, 20% sodium carbonate and Folin's reagent. After 2 h of incubation, absorbance at 760 nm was measured using a Biotek Epoch spectrophotometer, and polyphenol concentration was calculated using a catechin standard curve of known concentrations.

To determine the levels of IL-6 and advanced glycation end products (AGEs), two sandwich ELISA kits for rats were used (Elabscience Biotechnology CO., Ltd and Wuhan Fine Biotech Co., Ltd, respectively) following the manufacturers’ instructions. The reaction with samples produced a colour change, which was measured at 450 nm with the spectrophotometer (Biotek, Epoch).

### Liver MDA levels

MDA levels, as a final product of lipid peroxidation, were evaluated using a standardized colorimetric assay kit in the liver samples (Merck Life Science S.L.U., Madrid). The liver was homogenized in Tris–HCl 10 mM (pH 7.5) using a sample dispersing system (Ultra-Turrax® T10 Disperser, IKA, Staufen, Germany) and centrifuged at 9000 rpm for 10 min at 4ºC. Supernatants were collected and used for all the biochemical analyses. In brief, samples or standards were added to tubes containing 10.3 mM n-methyl-2-phenyl-indole in an acetonitrile mixture (3:1). Following this, 12 N HCl was added, and the samples were incubated for 1 h at 45 °C. Absorbance was measured at 586 nm. The concentration of MDA was determined by referencing a standard curve with known concentrations.

### Liver antioxidant enzyme activities

The liver was homogenized in Tris–HCl 10 mM (pH 7.5) and centrifuged at 10,000 rpm for 10 min, using the supernatant for analysis. Catalase (CAT), superoxide dismutase (SOD), and glutathione peroxidase (GPx) activities were measured in liver homogenates [33]. CAT activity was quantified spectrophotometrically by monitoring the breakdown of H2O2. SOD activity was determined using a xanthine/xanthine oxidase system, which generates the superoxide anion, leading to the reduction of cytochrome C that was measured at 550 nm. Activity of GPx was evaluated by an assay requiring H2O2 and NADPH, tracking the decrease in NADPH absorbance at 340 nm as it oxidizes to NADP +. Protein concentration was determined using a commercial kit (Merck Life Science S.L.U., Madrid, Spain) which was used to normalize the activity values. All enzyme activities were measured at 37 °C using a Shimadzu UV-2100 spectrophotometer (Shimadzu Corporation, Kyoto, Japan).

### Gene expression in liver

RNA levels for interleukin 6 (IL-6) and IL-10, carnitine palmitoyltransferase 1 (CPT-1), peroxisome proliferator-activated receptor alpha (PPARα), sterol regulatory element-binding protein 1 C (SREBP-1C), fatty acid synthase (FASN), and transforming growth factor beta (TGF-β) were measured using real-time polymerase chain reaction (RT-PCR). First, RNA was extracted from liver tissue (0.1 g) using Tripure® Isolation Reagent (Roche®, Basel, Switzerland) following the manufacturer’s instructions. Total RNA was quantified using the Take3 Microplate in a PowerwaveXS spectrophotometer (Biotek, Winooski, VT, USA). Subsequently, cDNA was synthesized from 1 µg of total RNA using 25 U MuLV reverse transcriptase in a mixture for retrotranscription (10 mM Tris–HCl, pH 9.0; 10 U RNase inhibitor; 0.1, 2.5 mM MgCl2; 50 mM KCl; 2.5 µM random hexamers; 500 µM of each dNTP). cDNA was diluted 1/10 and frozen at − 20ºC until analysis. Quantitative PCR was carried out on a GeneAmp® PCR System 9700 (Applied Biosystems, Madrid, Spain) (42ºC, 60 min). LightCycler rapid thermal cycler (Roche Diagnostics, Mannheim, Germany) was utilized for the RT-PCR using DNA-master SYBR Green. The amplification program consisted of a preincubation step for denaturation of template cDNA (95ºC, 10 min), then 45 cycles for denaturation, annealing, and an extension step. Fluorescence was measured at 72ºC after every cycle. The relative quantification was performed by standard calculations considering 2(-ΔΔCt). Gene expressions were normalized using β-actin as housekeeping after confirming its stable expression across experimental groups, and the primer specificity and amplification efficiency were assessed. Primer specificity was confirmed by melting curve analysis, which showed a single peak for each amplicon, indicating specific amplification. Amplification efficiency was calculated for each primer pair, yielding values close to 2.0 (corresponding to 100% efficiency), which is within the optimal range for qPCR assays. All samples were run in technical replicates. The primer sequences and temperature of annealing are described in the Table 2.

**Table 2:** Features of the used primers and amplification settings

**Gene**	**Fw sequence**	**Rev sequence**	**Annealing temperature**
Β-Actin	5'-AGG GAAATCGTGCGTGAC-3'	5'-CGCTCATTGCCGATAGTC-3'	95ºC for 15 s 60ºC for 30 s 72ºC 30 s
IL-6	5’-GCCACTGCCTTCCCTACTTCA-3’	5’-GACAGTGCATCGCTGTTCA-3’	95ºC for 15 s 60ºC for 30 s 72ºC for 30 s
IL-10	5’-GGCTCAGCACTGCTATGTTGCC-3’	5’-CACCAGTGATGATGCCATTCT-3’	95ºC for 15 s 60ºC for 30 s 72ºC for 30 s
CPT-1	5’-CTCCGCCTGAGCCATGAAG-3’	5’-CACCAGTGATGATGCCATTCT-3’	95ºC for 10 s 57ºC for 10 s 72ºC for 12 s
PPARα	5’-TGGAGTCCACGCATGTGAAG-3’	5’-CGCCAGCTTTAGCCGAATAG-3’	95ºC for 15 s 58ºC for 30 s 72ºC for 30 s
SREBP-1C	5’-AAACCTGAAGTGGTAGAAAC-3’	5’-TTATCCTCAAAGGCTGGG-3’	95ºC for 10 s 60ºC for 10 s 72ºC for 12 s
FASN	5’-AAAAGGAAAGTAGAGTGTGC-3’	5’-GACACATTCTGTTCACTACAG-3’	95ºC for 10 s 60ºC for 10 s 72ºC for 12 s
TGF-β	5’-GGAAATCAATGGGATCAGTC-3’	5’-CTGAAGCAGTAGTTGGTATC-3’	95ºC for 5 s 60ºC for 10 s 72ºC for 20 s

Abbreviations: Beta-Actin (β-Actin), (Interleukin 6 (IL-6), Interleukin 10 (IL-10), Carnitine palmitoyltransferase 1 (CPT-1), Peroxisome proliferator-activated receptor alpha (PPARα), Sterol regulatory element-binding protein 1 C (SREBP-1C), Fatty acid synthase (FASN), Transforming growth factor beta (TGF-β).

### Statistical analysis

Graph Pad Prism 8.3.0 was used for the statistical analysis. Data are represented as the mean ± standard error of the mean (SEM) with p < 0.05 to be statistically significant. The normal distribution of the data was assessed by the Shapiro–Wilk test. One-way analysis of variance (ANOVA) was used to evaluate the data. When significant differences were found, a post hoc Least Significant Difference (LSD) test was used to establish differences between the different data.

## Results

### Body weight

**Fig. 2:**
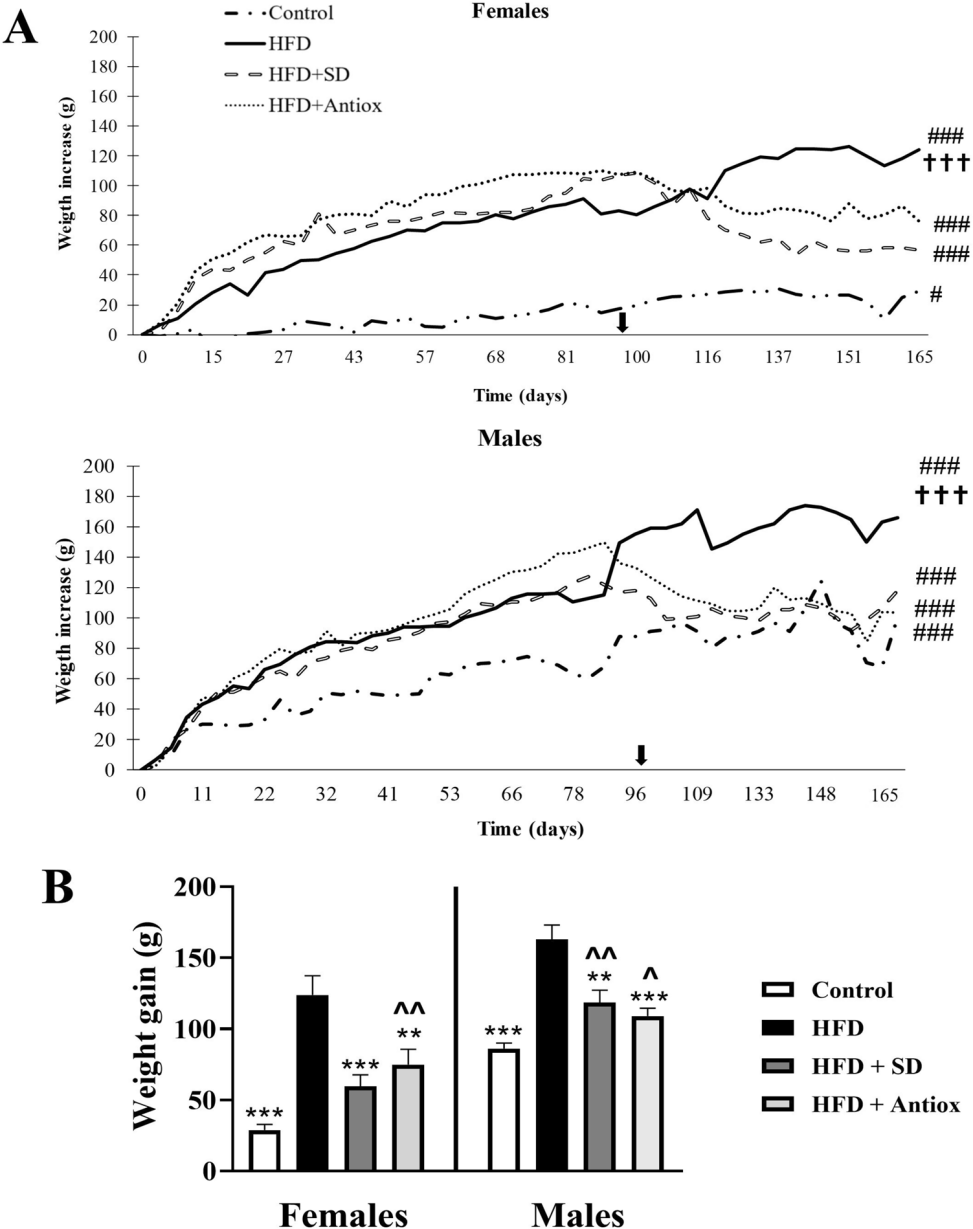
A/Body weight (g) evolution of both sex rats (n = 7/sex and group) of the different interventional groups from the beginning of the study (day 0) until the last day of study. The arrow indicates the moment in which the diet was changed. B/Body weight gain (g) of female and male rats as the difference between the final weight and the initial weight at the beginning of the experiment. One-way ANOVA and LSD post hoc were used for differences between last and first day for every group (A) and at the end of the study between experimental groups (B). (A): # p-value < 0.05 and ### p-value < 0.001 indicate differences between the last day respect to the day 0; ✝✝✝ p-value < 0.001 indicates differences between the last day along the groups; (B): ** p-value < 0.01 and *** p-value < 0.001 respect to HFD group; Λ p-value < 0.05 and ΛΛ p-value < 0.01 indicates differences respect to the control group. Antiox: antioxidants; HFD: high fat diet; SD: standard diet

The increase in weight gain of both female and male rats throughout the experimental procedure is represented in Fig. 2. The initial weight for females was 249.5 ± 2.1, 226.9 ± 5.7, 234.7 ± 4.3, and 224.7 ± 6.7 for control, HFD, HFD + SD, and HFD + Antiox, respectively. The initial weight for males was 392.9 ± 15.3, 396.7 ± 8.3, 369.6 ± 10.8, and 360.5 ± 9.1 for control, HFD, HFD + SD, and HFD + Antiox, respectively. As expected, an increase in weight at the end of the 3-month diet intervention was observed in all the groups with respect to the beginning, although the animals that followed a HFD during the full 5 months reached the highest body weight gain in both sexes compared with the first day of the study. At the end of the diet change (after 2 additional months), the animals that continued for 2 more months more with an HFD presented statistically higher weight gain in comparison with all the other groups (control group and the two groups that followed a HFD for three months and changed to a standard diet for the following two months) (p < 0.001, Table S1). Greater variations in weight gain were observed among the female intervention groups, with those switched to a standard or antioxidant-rich diet still showing higher gains than control females (that followed a standard diet during the 5 months). The transition to a healthier diet was not related to a reduced food intake as all the animals consumed all the provided food.

### Lipid deposits analysis in liver

**Fig. 3:**
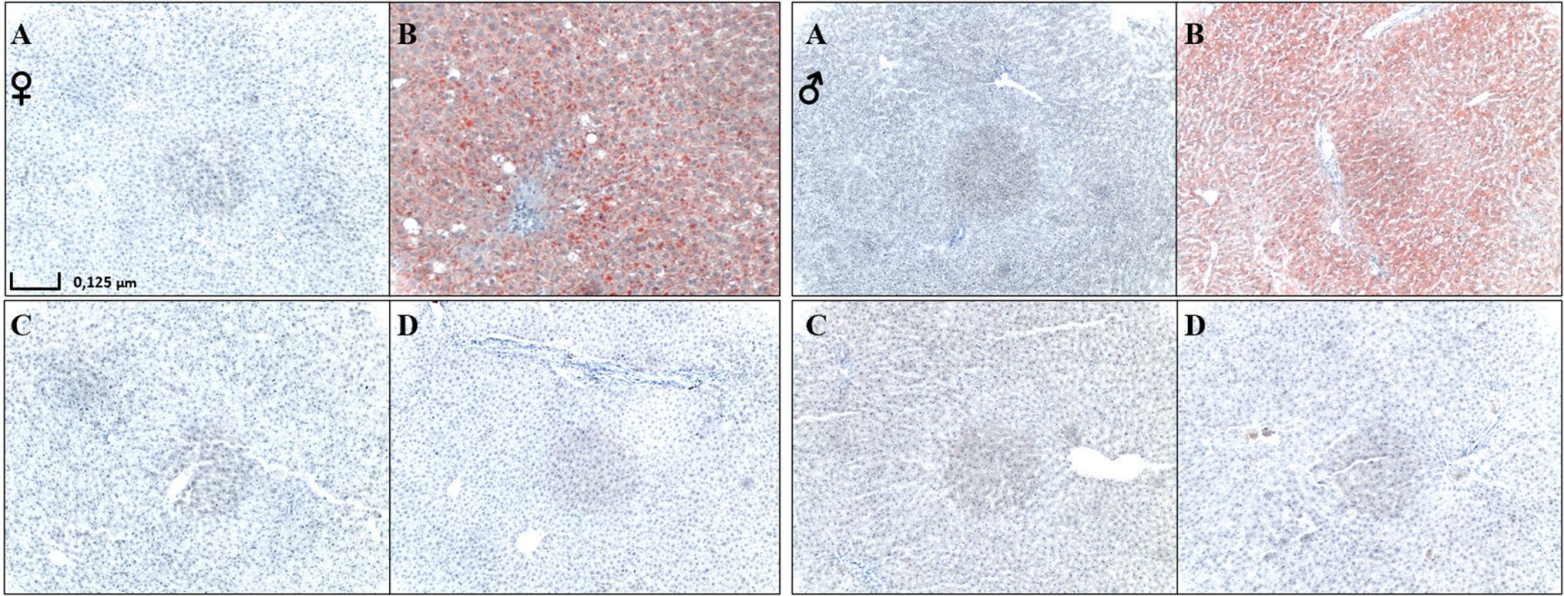
Representative histological sections of liver from the rats stained with Oil Red. Four images on the left for females, and four on the right for males. A: Control, B: high fat diet (HFD), C: HFD + Standard diet (HFD + SD), and D: HFD + Antioxidants (HFD + Antiox)

Liver lipid deposit analysis using immunohistochemical Oil Red staining revealed that animals from both sexes that followed an HFD for 5 months presented a great lipid accumulation, that was absent in control animals. This hepatic lipid accumulation was drastically reduced in animals that switched to either a standard or an antioxidant-rich diet; in a similar manner in both sexes (Fig. 3 and Table 3).

**Table 3:** Quantification of lipid accumulation in hepatic tissue

Component	Female	Male
Control	0.00 ± 0.00	0.00 ± 0.00
High-fat diet (HFD)	9.41 ± 0.44*	10.54 ± 0.20*
HFD- standard diet	0.00 ± 0.00	0.07 ± 0.05
HFD-antioxidant diet	0.30 ± 0.05	0.02 ± 0.01

Data represent the mean ± SEM of the proportion of red stained area (oil red staining) from 4 liver images (10 × magnification) from 4 animals per group and sex. One-way ANOVA and LSD post hoc. * p-value < 0.01 respect to control group.

### Plasma polyphenolic concentration

**Fig. 4:**
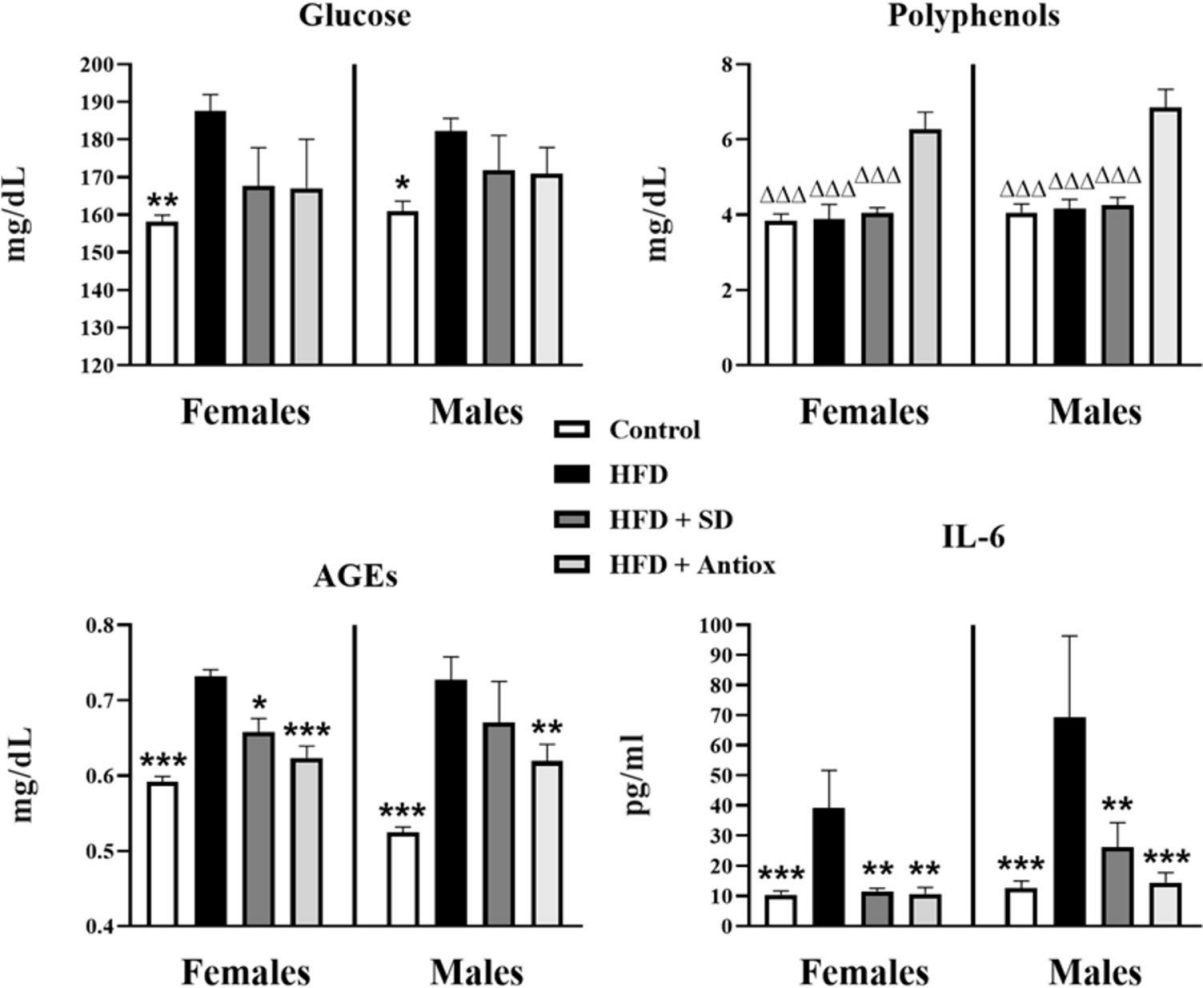
Plasma levels of glucose, advanced glycation end products (AGEs), polyphenolic compounds, and IL-6 of both female and male rats (n = 7/sex and group) for the groups control, high fat diet (HFD), and HFD with change to standard (HFD + SD) or antioxidant-rich (HFD + Antiox) diets. One-way ANOVA and LSD post hoc. * p-value < 0.05, ** p-value < 0.01 and *** p-value < 0.001 respect to HFD group; ΔΔΔ p-value < 0.001 respect to HFD + Antiox group

The analysis in plasma of the rats showed statistically significant higher concentrations of polyphenolic content (expressed as ng/L of plasma, Fig. 4) in the animals that were fed with a diet rich in antioxidants (3.88 ± 0.40 for females, 4.15 ± 0.26 for males) when compared with all other groups (for females: 3.84 ± 0.15, 4.05 ± 0.14, 6.28 ± 0.46; for males: 4.06 ± 0.23, 4.56 ± 0.30, 6.85 ± 0.49; for control, HFD + SD and HFD + Antiox, respectively; p-values < 0.001) (Table S1).

### Plasma glucose and AGEs levels

Figure 4 also shows the plasmatic glucose and AGEs levels found in the different studied groups for both sexes. The HFD group presented the highest values for both parameters with statistical differences with respect to the respective controls in both sexes. For glucose, after intervention with both diets (standard and rich in antioxidants) a tendency to recover the control levels was observed in both female and male animals. The levels of AGEs in plasma followed the same pattern though showing statistically significant differences with respect to the HFD group (for females, p < 0.001, p < 0.05 and p < 0.01 for control, HFD + SD, and HFD + Antiox, respectively; for males, p < 0.001 and p < 0.01 for control and HFD + Antiox, respectively) (Table S1).

### Plasma pro-inflammatory marker

In Fig. 4 the plasma levels of the pro-inflammatory cytokine IL-6 are also represented. The groups fed with HFD presented the highest values for this cytokine in both sexes compared with control group fed a standard diet (p < 0.001, Table S1). Moreover, animals from both sexes that switched to standard diet (p < 0.05) or antioxidant diet (p < 0.05 for females and p < 0.001 for males, Table S1) during the last two months presented plasma levels similar to control animals for this cytokine.

### Oxidative-related markers in liver

**Fig. 5:**
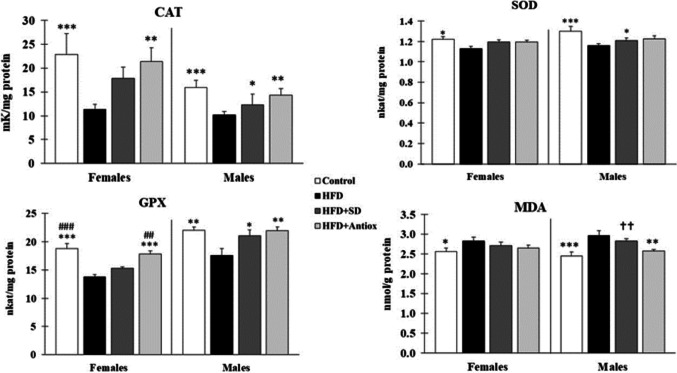
Catalase (CAT), superoxide dismutase (SOD), glutathione peroxidase (GPX) activities and malondialdehyde (MDA) levels in both female and male rats (n = 7/sex and group) from the liver for the groups control, high fat diet (HFD), and HFD with change to standard (HFD + SD) or antioxidant-rich (HFD + Antiox) diets. One-way ANOVA and LSD post hoc. * p-value < 0.05, ** p-value < 0.01 and *** p-value < 0.001 respect to HFD group; ## p-value < 0.01 and ### p-value < 0.001 respect to HFD + SD group; ✝✝ p-value < 0.01 respect to control group

The observed changes in the antioxidant enzyme activities and the MDA levels in liver are represented in Fig. 5 and Table S1. Catalase activity in the HFD group was lower compared with the other groups, with statistically significant differences in females compared with the control and HFD + antiox groups (p < 0.001 and p < 0.01, respectively). In males, significant differences were found compared with the other three groups (p < 0.001 for control, p < 0.05 for HFD + SD, p < 0.01 for HFD + Antiox). SOD activity was higher in the control group for both sexes compared with the HFD (p < 0.05 for females, p < 0.001 for males). Also, HFD + SD was higher in males compared with the HFD group (p < 0.05). GPX activity was also lower in the HFD group in both sexes compared with the control (p < 0.001 for females, p < 0.01 for males) and HFD + Antiox (p < 0.001 for females, p < 0.01 for males) groups. For males, HFD + SD presented higher values compared with the HFD group (p < 0.05). In addition, the antioxidant-rich diet group in females showed higher GPX values with respect to the HFD + SD (p < 0.01). MDA levels were higher in the HFD group compared with the control in both sexes with significant differences (p < 0.05 for females, p < 0.001 for males). Additionally, HFD was significantly different from HFD + Antiox in males (p < 0.01). Only in males, MDA levels in the HFD + SD group were higher compared with the control (p < 0.01).

### Gene expression

**Fig. 6:**
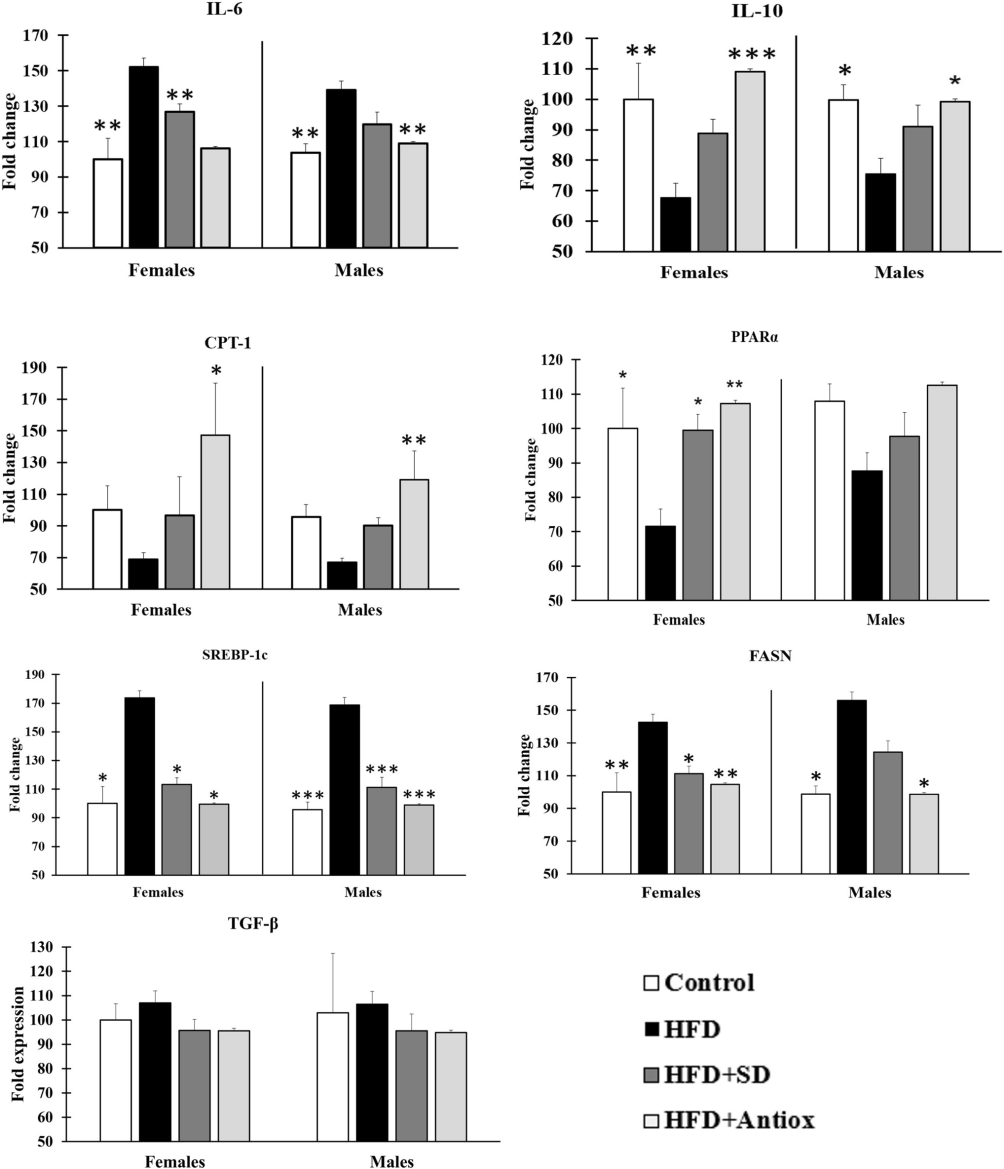
Gene expression of IL-6 and IL-10, CPT-1, PPARα, SREBP-1C, FASN and TGF-β in both female and male rats (n = 7/sex and group) from the liver for the groups control, high fat diet (HFD), and HFD with change to standard (HFD + SD) or antioxidant-rich (HFD + Antiox) diets. One-way ANOVA and LSD post hoc. * p-value < 0.05, ** p-value < 0.01 and *** p-value < 0.001 respect to HFD group

Gene expressions related to the inflammatory status (IL-6 and IL-10) and lipid metabolism (CPT-1, PPARα, SREBP-1c, and FASN) in the liver for the different studied groups for both sexes are represented in Fig. 6 and Table S1. The continuous ingestion of an HFD for 5 months significantly increased gene expression of the pro-inflammatory IL-6 and decreased that of the anti-inflammatory IL-10 in liver from both female and male rats. The change to a standard diet partly reverted these changes, which were fully reversed in animals that switched to antioxidant-rich diet, reaching cytokine values similar to those of the respective controls in both females and males. Significant differences in CPT-1 expression were observed between the HFD and HFD + Antiox groups in both females (p < 0.05) and males (p < 0.01). For PPARα, significant differences between the HFD and other groups were found only in females (p < 0.05 with respect to control and HFD + SD, p < 0.01 with respect to HFD + Antiox), although male animals showed a similar profile. Finally, the 5-month HFD statistically increased the expression of SREBP-1c (p < 0.05 for females, p < 0.001 for males) and FASN (p < 0.05 for HFD + SD and p < 0.01 for control and HFD + Antiox in the female groups; p < 0.05 for control and HFD + Antiox in males) compared with the other groups, except for FASN expression in HFD + SD group for males.

Figure 6 also shows the gene expression of TGF-β as a biomarker in the development of hepatic fibrosis. The results indicated no significant differences between the interventional groups in either females or males.

## Discussion

Over the decades, research has established the influence of diet on the human body. Currently, malnutrition is one of the biggest health problems in developed countries [1]. Excessive caloric intake can lead to fat accumulation in the liver, promoting MAFLD. This syndrome is characterized by a network of metabolic alterations for which there are limited specific treatments [3, 19]. Different studies have been focused on evaluating the effects of a high-fat or high-glucose diet in MALFD models [34, 35] and the effects that specific compounds with anti-inflammatory and/or antioxidant capacities, including polyphenols found in fruits and vegetables, can exert [13, 26, 36, 37]. However, fewer studies have evaluated the effects of a complete dietary change within the same animals, as most compare different groups without dietary changes in the same group. The current study reproduced the pathology in a middle-aged rat model in both sexes, showing improvements in histology, plasma markers, and hepatic oxidative stress markers following dietary intervention, including both standard and antioxidant-rich diets. These effects were more pronounced in the groups consuming antioxidant-rich diets, indicating that a higher intake of bioactive compounds with antioxidant and anti-inflammatory potential can enhance the reversal of the hepatic alterations in both female and male rats. These findings may be related to the higher content of fatty acids, cholesterol, and carbohydrates in the HFD compared with the antioxidant diet.

The HFD group showed greater weight gain compared with the other groups; switching to a healthy diet resulted in a less pronounced increase in the body weight gain, in both female and male animals. Previous studies have reported similar changes in body weight, demonstrating that excessive fat intake induced excess weight in rats [38–40]. This excess weight was also reflected in the lipid deposits observed in the liver sections. No lipid deposits were observed in the control groups, but the HFD groups showed significant lipid accumulation. In contrast, animals that modified their diet to a healthy one showed no staining at the end of the study in either sex. These results, consistent with other studies [41, 42], suggest that the HFD induced significant hepatic lipid accumulation, aligning with the MAFLD pathogenesis. The absence of lipid accumulation in the control and in diet-changed groups indicated that adequate dietary intervention could prevent hepatic steatosis. These findings support the hypothesis that diet plays a crucial role in regulating hepatic lipid metabolism and suggest that dietary management may help prevent or reverse MAFLD. Similar effects have been reported with antioxidant interventions in HFD models, reporting a reduction in hepatic lipid content [43, 44]. Furthermore, the similar responses observed in females and males across the different groups suggest that the effect of diet on hepatic lipid accumulation did not differ markedly between sexes in this experimental model. These outcomes indicate that diet may act as a sex-independent factor in the development of metabolic fatty liver disease.

Glucose and AGEs levels followed a similar pattern across the groups, with both females and males in the HFD group showing elevated levels when compared with the other groups. Consistent with previous studies, the HFD induced insulin resistance, leading to elevated blood glucose levels [45, 46]. Excessive saturated fat intake disrupts insulin signalling and impairs glucose uptake, while a balanced diet with lower fat content enhances insulin sensitivity, reducing blood glucose levels [47]. Moreover, the diets rich in antioxidants help maintain glucose control by improving insulin sensitivity and mitigating oxidative stress and inflammation, which negatively impact glucose regulation [48]. The elevated glucose in the HFD group also promoted the formation of AGEs, compounds generated through the glycation of proteins or lipids, that accumulate and damage tissues [49, 50]. Reducing lipid and glucose levels through diet can decrease AGE formation [51]. In MAFLD, hyperglycaemia and insulin resistance contribute to increased triglyceride synthesis in the liver, promoting steatosis and fibrosis, while AGEs further exacerbate damage, inflammation, and fibrosis [52]. In the present study, the used diets (specially antioxidant-rich one) reduced AGE levels close to the control group, in both females and males.

An HFD also contributes to chronic low-grade inflammation. Excess fat accumulation, especially saturated fats, can activate the immune system and trigger an inflammatory response [53]. IL-6 is a key cytokine in this process. The increase in IL-6 levels reflects an inflammatory activation, which is associated with insulin resistance and progression of metabolic diseases, such as MAFLD [54]. This is consistent with the observed hepatic upregulation of the IL-6 gene expression and its increased plasma levels after an HFD in the current work. However, the diet intervention lowered the levels of this inflammatory cytokine, indicating decreased systemic inflammation and reflecting the effect of a healthier diet in reducing chronic inflammation [55]. Moreover, the decrease of IL-10 gene expression following an HFD could be related to anti-inflammatory regulation, as IL-10 inhibits pro-inflammatory cytokines production. Previous studies also shown reduced IL-10 levels after an HFD [56, 57]. This may be linked to the deregulation of T cells function, a key source of this cytokine. The IL-10 reduction could reflect immune imbalance and disruption of anti-inflammatory pathways [58, 59]. Although both dietary interventions improved the inflammatory parameters in our work, the antioxidant-rich diet seemed to have a stronger effect on them in both female and male rats. It may be due to the anti-inflammatory properties of components such as polyphenols. These findings align with previous works using different isolated polyphenolic compounds [26, 37].

The activity of the hepatic antioxidant enzymes, together with lipid peroxidation levels, supports the detrimental effects of an HFD. CAT, SOD and GPx activities were consistently lower in the HFD group compared with the other groups in both sexes, indicating a reduced antioxidant defence capacity. Other studies have also reported similar reductions in CAT levels in fatty liver rat models [45, 60]. This enzyme converts hydrogen peroxide, a toxic byproduct of superoxide dismutation, into water and oxygen. Thus, its reduction suggests a decreased capacity to neutralize ROS in the HFD group. SOD catalyses the conversion of the highly reactive superoxide anion into hydrogen peroxide and oxygen, while GPx reduces hydrogen peroxide and lipid hydroperoxides to water and non-reactive lipids using reduced glutathione. GPx plays a key role in protecting cell membranes from lipid peroxidation, which can impair liver cell integrity and function [61]. Similarly, lower SOD and GPx activities reflect a weakened antioxidant defence system in this group, as previously described [38, 45, 62]. In the current work, dietary intervention restored antioxidant capacity, particularly in the antioxidant-rich group, which significantly increased CAT and GPx activity and showed a tendency to increase SOD values when compared with the HFD group. Moreover, it yielded better results than the standard diet in groups switching from HFD to a balanced diet in both sexes, although without statistical significance. Other studies showed similar results, where different antioxidant extracts reduced oxidative stress [63, 64]. Consistent with the antioxidant enzyme results, the HFD group showed the highest MDA levels, which were reversed after dietary interventions. MDA is a key marker of lipid peroxidation and oxidative damage, and its elevated levels are associated with metabolic diseases [65]. These findings indicate that HFD reduced antioxidant activity compared with the healthy diets, leading to increased MDA levels with significant cellular effects. Similar results have been reported in studies in which an HFD or a high-fat emulsion increased MDA concentrations [38, 66, 67]. In contrast, some works observed increased enzymatic activity in a high-fat liver mouse model [68]; however, these differences may be due to shorter intervention periods (2 months *vs* 5 months in this study) and the use of a high-fat, high-fructose diet. Additionally, antioxidant-rich foods have been shown to reduce MDA levels compared with exclusively high-fat diets [69]. In the present work, dietary changes reversed MDA levels, with a greater reduction observed in the antioxidant diet. This highlights its capacity to mitigate HFD-induced damage, likely due to the antioxidant and anti-inflammatory effects of compounds such as polyphenols [13].

PPARα and CPT-1 are involved in fatty acid metabolism. PPARα is a transcriptional regulator that promotes the increase of malonyl-CoA decarboxylase, leading to the upregulation of CPT-1 [70, 71]. CPT-1 is responsible for beta-oxidation by binding to long-chain fatty acids and combining them with carnitine, forming a complex that can cross the outer mitochondrial membrane to be oxidized [72]. This process is key for fatty acid oxidation and energy production in cells. Under HFD conditions, their function can be disrupted due to the excess of fatty acids and other lipids, leading to dysfunction of beta-oxidation regulation [73]. In fatty liver disease, lower expression of CPT-1 and PPARα limits the ability to manage excess of fat [74]. The current results showed a decrease in both CPT-1 and PPARα gene expression in the HFD group, with a significant increase when the diet was changed to an antioxidant-rich one, and a slight increase in the group that switched to a standard diet. Thus, the consumption of antioxidants, such as polyphenols, may appear to better counteract the effects of a diet rich in fats. Oxidative stress can impair mitochondria and other cellular components, disrupting the regulation of proteins like PPARα and CPT-1 [74]. By reducing the accumulation of free radicals and inflammation, polyphenols may create a more favourable environment for the activation of PPARα, and consequently the CPT-1 increase, contributing to an improved beta-oxidation in the animals eating a diet rich in antioxidants [75, 76]. In fact, polyphenols possess antioxidant and anti-inflammatory properties that could help mitigate oxidative stress induced by an HFD [36]. Additionally, this situation can be enhanced by the action of some central genes related to lipogenesis. SREBP-1c is a transcription factor that upregulates lipogenic genes, including FASN, which is directly responsible for synthesizing long-chain fatty acids from acetyl-CoA [77]. The HFD in our work upregulated SREBP-1c that could trigger an increased FASN expression. Overall it can lead to enhanced lipogenesis and the hepatic triglyceride accumulation [78]. This may contribute to the development of MAFLD and other metabolic disorders [77]. However, the antioxidant-rich diet notably downregulated both SREBP-1c and FASN expressions. Polyphenols are known to inhibit lipogenic pathways by reducing the activation of SREBP-1c, thus lowering the synthesis of fatty acids [79, 80]. This reduction in SREBP-1c and FASN can help mitigate the harmful effects of excessive lipid accumulation caused by an HFD. In fact, the antioxidant properties of polyphenols play a role in decreasing oxidative stress and inflammation, which are key factors in the activation of SREBP-1c [80]. Interestingly, these transcriptional genes responses were equal in both females and males indicating that the dietary approach could be a broad strategy for both sexes in MAFLD.

Although the fatty liver development was confirmed in the HFD by both histological analyses and molecular results, TGF-β levels were maintained unchanged. This marker is associated with liver damage and fibrosis. In this context, TGF-β acts as a key mediator in cellular signalling, facilitating the activation of hepatic stellate cells (HSCs) and promoting the deposition of extracellular matrix, that leads to scar tissue formation [81, 82]. In the current work, it is possible that the HFD caused lipid deposits in both sexes but without triggering full activation of the fibrogenic cycle. It could be possible that the duration of the HFD was not enough to induce the full fibrotic process. However, other works showed that antioxidant intake was effective in preventing TGF-β activation, mainly by inhibiting ROS production, which amplify profibrotic signalling. For example, a study with a maleic acid derivative demonstrated that specific antioxidants could suppress HSC activation and reduce fibrosis markers in TGF-β-treated mouse model [83].

## Conclusion

In conclusion, this study provides insight into the harmful effects that an HFD can have in both sexes, in terms of oxidative stress and inflammation, and the alterations induced by HFD in liver function and fatty acid metabolism in both female and male rats. Furthermore, this work demonstrates the potential to reverse these hepatic alterations through dietary changes, with similar effects in females and males. Notably, the intake of a diet rich in compounds with antioxidant and anti-inflammatory properties resulted in a greater improvement in the related biomarkers even in comparison with a standard diet. In addition, the results support the relevance of dietary interventions in a MAFLD model in both sexes, as not all the studies focused on the sex responses, or they lacked within-subject dietary interventions. Despite the progress in the pharmacological treatment of MAFLD, lifestyle modifications remain an essential aspect of its managing. Therefore, more studies using animal models of fatty liver, followed by dietary interventions, are needed to deepen the understanding of the underlying mechanisms, with particular attention to sex differences.

## Supplementary Information

Below is the link to the electronic supplementary material.

Supplementary file1 (JPEG 5850 KB)

Supplementary file2 (JPEG 6306 KB)

Supplementary file3 (JPG 374 KB)

Supplementary file4 (JPEG 5838 KB)

Supplementary file5 (JPEG 5488 KB)

Supplementary file6 (JPEG 5647 KB)

Supplementary file7 (JPEG 6012 KB)

Supplementary file8 (JPEG 6037 KB)

Supplementary file9 (DOCX 24 KB)

## Data Availability

Data is provided within the manuscript.

## References

[1] Kliemann N, Al Nahas A, Vamos EP, Touvier M, Kesse-Guyot E, Gunter MJ, Millett C, Huybrechts I (2022) Ultra-processed foods and cancer risk: from global food systems to individual exposures and mechanisms. Br J Cancer 127:14–20. 10.1038/s41416-022-01749-y 35236935 10.1038/s41416-022-01749-y PMC9276654

[2] Kobylinska M, Antosik K, Decyk A, Kurowska K (2022) Malnutrition in Obesity: Is It Possible? Obes Facts 15:19–25. 10.1159/000519503 34749356 10.1159/000519503 PMC8820192

[3] Perdomo CM, Fruhbeck G, Escalada J (2019) Impact of Nutritional Changes on Nonalcoholic Fatty Liver Disease. Nutrients 11(3):677. 10.3390/nu11030677 30901929 10.3390/nu11030677 PMC6470750

[4] Eslam M, Sanyal AJ, George J, Sanyal A, Neuschwander-Tetri B, Tiribelli C, Kleiner DE, Brunt E, Bugianesi E, Yki-Järvinen H, Grønbæk H (2020) MAFLD: A Consensus-Driven Proposed Nomenclature for Metabolic Associated Fatty Liver Disease. Gastroenterology 158:1999–2014. 10.1053/j.gastro.2019.11.312 32044314 10.1053/j.gastro.2019.11.312

[5] Younossi ZM, Golabi P, Paik JM, Henry A, Van Dongen C, Henry L (2023) The global epidemiology of nonalcoholic fatty liver disease (NAFLD) and nonalcoholic steatohepatitis (NASH): a systematic review. Hepatology 77:1335–1347. 10.1097/HEP.0000000000000004 36626630 10.1097/HEP.0000000000000004 PMC10026948

[6] Lim GEH, Tang A, Ng CH, Chin YH, Lim WH, Tan DJH, Yong JN, Xiao J, Lee CW, Chan M et al (2023) An Observational Data Meta-analysis on the Differences in Prevalence and Risk Factors Between MAFLD vs NAFLD. Clin Gastroenterol Hepatol 21(619–629):e617. 10.3748/wjg.v30.i27.3273 10.1016/j.cgh.2021.11.038 34871813

[7] Riazi K, Azhari H, Charette JH, Underwood FE, King JA, Afshar EE, Swain MG, Congly SE, Kaplan GG, Shaheen AA (2022) The prevalence and incidence of NAFLD worldwide: a systematic review and meta-analysis. Lancet Gastroenterol Hepatol 7:851–861. 10.1016/S2468-1253(22)00165-0 35798021 10.1016/S2468-1253(22)00165-0

[8] Fan J, Luo S, Ye Y, Ju J, Zhang Z, Liu L, Yang J, Xia M (2021) Prevalence and risk factors of metabolic associated fatty liver disease in the contemporary South China population. Nutr Metab (Lond) 18:82. 10.1186/s12986-021-00611-x 34496912 10.1186/s12986-021-00611-x PMC8425111

[9] Wu S, Li Y, Zhang Y, Su X, Zuo Y, Chen G, Xu G, Chen S, He Y, Wang A (2024) Sex and age differences in the association between metabolic dysfunction-associated fatty liver disease and heart failure: a prospective cohort study. Circ Heart Fail 17:e010841. 10.1161/circheartfailure.123.010841 38348678 10.1161/CIRCHEARTFAILURE.123.010841

[10] Francque SM, Marchesini G, Kautz A, Walmsley M, Dorner R, Lazarus JV, Zelber-Sagi S, Hallsworth K, Busetto L, Fruhbeck G et al (2021) Non-alcoholic fatty liver disease: a patient guideline. JHEP Rep 3:100322. 10.1016/j.jhepr.2021.100322 34693236 10.1016/j.jhepr.2021.100322 PMC8514420

[11] Heeren J, Scheja L (2021) Metabolic-associated fatty liver disease and lipoprotein metabolism. Mol Metab 50:101238. 10.1016/j.molmet.2021.101238 33892169 10.1016/j.molmet.2021.101238 PMC8324684

[12] Sakurai Y, Kubota N, Yamauchi T, Kadowaki T (2021) Role of Insulin Resistance in MAFLD. Int J Mol Sci 22:4156. 10.3390/ijms22084156 33923817 10.3390/ijms22084156 PMC8072900

[13] Tejada S, Capo X, Mascaro CM, Monserrat-Mesquida M, Quetglas-Llabres MM, Pons A, Tur JA, Sureda A (2021) Hepatoprotective Effects of Resveratrol in Non-Alcoholic Fatty Live Disease. Curr Pharm Des 27:2558–2570. 10.2174/1381612826666200417165801 32303170 10.2174/1381612826666200417165801

[14] Clare K, Dillon JF, Brennan PN (2022) Reactive oxygen species and oxidative stress in the pathogenesis of MAFLD. J Clin Transl Hepatol 10:939–946. 10.14218/JCTH.2022.00067 36304513 10.14218/JCTH.2022.00067 PMC9547261

[15] Quetglas-Llabres MM, Monserrat-Mesquida M, Bouzas C, Garcia S, Mateos D, Casares M, Gomez C, Ugarriza L, Tur JA, Sureda A (2024) Effects of a Two-Year Lifestyle Intervention on Intrahepatic Fat Reduction and Renal Health: Mitigation of Inflammation and Oxidative Stress, a Randomized Trial. Antioxidants 13(7):754. 10.3390/antiox13070754 39061823 10.3390/antiox13070754 PMC11273830

[16] Chan WK, Chuah KH, Rajaram RB, Lim LL, Ratnasingam J, Vethakkan SR (2023) Metabolic dysfunction-associated steatotic liver disease (MASLD): a state-of-the-art review. J Obes Metab Syndr 32:197–213. 10.7570/jomes23052 37700494 10.7570/jomes23052 PMC10583766

[17] Administration FAD (2024) FDA Approves First Treatment for Patients with Liver Scarring Due to Fatty Liver Disease. In. U.S. Food and Drug Administration. Retrieved from https://www.fda.gov/news-events/press-announcements/fda-approves-first-treatment-patients-liver-scarring-due-fatty-liver-disease. Accessed November 8, 2024

[18] Kim DG, Krenz A, Toussaint LE, Maurer KJ, Robinson SA, Yan A, Torres L, Bynoe MS (2016) Non-alcoholic fatty liver disease induces signs of Alzheimer’s disease (AD) in wild-type mice and accelerates pathological signs of AD in an AD model. J Neuroinflammation 13:1. 10.1186/s12974-015-0467-5 26728181 10.1186/s12974-015-0467-5 PMC4700622

[19] Long MT, Noureddin M, Lim JK (2022) AGA Clinical Practice Update: Diagnosis and Management of Nonalcoholic Fatty Liver Disease in Lean Individuals: Expert Review. Gastroenterology 163(764–774):e761. 10.1053/j.gastro.2022.06.023 10.1053/j.gastro.2022.06.023 PMC9398982 35842345

[20] Quetglas-Llabres MM, Monserrat-Mesquida M, Bouzas C, Llompart I, Mateos D, Casares M, Ugarriza L, Martinez JA, Tur JA, Sureda A (2023) Mediterranean Diet Improves Plasma Biomarkers Related to Oxidative Stress and Inflammatory Process in Patients with Non-Alcoholic Fatty Liver Disease. Antioxidants 12(4):833. 10.3390/antiox12040833 37107208 10.3390/antiox12040833 PMC10134978

[21] Karimi M, Akhgarjand C, Houjaghani H, Nejad MM, Sohrabpour AA, Poustchi H, Mohammadi H, Chamari M, Imani H (2025) The effect of intermittent fasting diet in comparison with low-calorie diet on inflammation, lipid profile, glycemic index, liver fibrosis in patients with metabolic-associated fatty liver disease (MAFLD): a randomized controlled trial. Clin Ther 47:e9–e16. 10.1016/j.clinthera.2025.01.007 39915199 10.1016/j.clinthera.2025.01.007

[22] Ortenzi VH, Oliveira AC, Vasconcelos RP, Neves MB, Teixeira AJ, Oliveira KA, Ferreira ACF, Takiya CM, Fortunato RS (2024) High-fat diet elicits sex-based differences in liver inflammatory cytokines and redox homeostasis. Appl Physiol Nutr Metab 49:1083–1092. 10.1139/apnm-2023-0457 38648669 10.1139/apnm-2023-0457

[23] Bentanachs R, Blanco L, Montesinos M, Sala-Vila A, Lazaro I, Rodriguez-Morato J, Sanchez RM, Laguna JC, Roglans N, Alegret M (2023) Adipose Tissue Protects against Hepatic Steatosis in Male Rats Fed a High-Fat Diet plus Liquid Fructose: Sex-Related Differences. Nutrients 15(18):3909. 10.3390/nu15183909 37764693 10.3390/nu15183909 PMC10534325

[24] Yao W, Fan M, Qian H, Li Y, Wang L (2024) Quinoa polyphenol extract alleviates non-alcoholic fatty liver disease via inhibiting lipid accumulation, inflammation and oxidative stress. Nutrients. 10.3390/nu16142276 39064719 10.3390/nu16142276 PMC11279623

[25] Sheng X, Zhan P, Wang P, He W, Tian H (2024) Mitigation of high-fat diet-induced hepatic steatosis by thyme (*Thymus quinquecostatus* Celak) polyphenol-rich extract (TPE): insights into gut microbiota modulation and bile acid metabolism. Food Funct 15:7333–7347. 10.1039/D3FO05235D 38305590 10.1039/d3fo05235d

[26] Kessoku T, Imajo K, Honda Y, Kato T, Ogawa Y, Tomeno W, Kato S, Mawatari H, Fujita K, Yoneda M et al (2016) Resveratrol ameliorates fibrosis and inflammation in a mouse model of nonalcoholic steatohepatitis. Sci Rep 6:22251. 10.1038/srep22251 26911834 10.1038/srep22251 PMC4766502

[27] Liu YL, Zhang QZ, Wang YR, Fu LN, Han JS, Zhang J, Wang BM (2020) Astragaloside IV improves high-fat diet-induced hepatic steatosis in nonalcoholic fatty liver disease rats by regulating inflammatory factors level via TLR4/NF-kappaB signaling pathway. Front Pharmacol 11:605064. 10.3389/fphar.2020.605064 33708118 10.3389/fphar.2020.605064 PMC7941269

[28] Spencer JP (2010) The impact of fruit flavonoids on memory and cognition. Br J Nutr 104(Suppl 3):S40-47. 10.1017/s0007114510003934 20955649 10.1017/S0007114510003934

[29] Sampey BP, Vanhoose AM, Winfield HM, Freemerman AJ, Muehlbauer MJ, Fueger PT, Newgard CB, Makowski L (2011) Cafeteria diet is a robust model of human metabolic syndrome with liver and adipose inflammation: comparison to high-fat diet. Obesity 19:1109–1117. 10.1038/oby.2011.18 21331068 10.1038/oby.2011.18 PMC3130193

[30] Ramis MR, Sarubbo F, Moranta D, Tejada S, Llado J, Miralles A, Esteban S (2020) Cognitive and Neurochemical Changes Following Polyphenol-Enriched Diet in Rats. Nutrients 13(1):59. 10.3390/nu13010059 33375450 10.3390/nu13010059 PMC7824548

[31] Jia Q, Cao H, Shen D, Li S, Yan L, Chen C, Xing S, Dou F (2019) Quercetin protects against atherosclerosis by regulating the expression of PCSK9, CD36, PPARgamma, LXRalpha and ABCA1. Int J Mol Med 44:893–902. 10.3892/ijmm.2019.4263 31524223 10.3892/ijmm.2019.4263 PMC6658003

[32] Kinkel AD, Fernyhough ME, Helterline DL, Vierck JL, Oberg KS, Vance TJ, Hausman GJ, Hill RA, Dodson MV (2004) Oil red-O stains non-adipogenic cells: a precautionary note. Cytotechnology 46:49–56. 10.1007/s10616-004-3903-4 19003258 10.1007/s10616-004-3903-4 PMC3449473

[33] Sureda A, Batle JM, Tauler P, Aguilo A, Cases N, Tur JA, Pons A (2004) Hypoxia/reoxygenation and vitamin C intake influence NO synthesis and antioxidant defenses of neutrophils. Free Radic Biol Med 37:1744–1755. 10.1016/j.freeradbiomed.2004.07.033 15528034 10.1016/j.freeradbiomed.2004.07.033

[34] Li X, Lu Y, Liang X, Zhou X, Li D, Zhang Z, Niu Y, Liu S, Ye L, Zhang R (2023) A new NASH model in aged mice with rapid progression of steatohepatitis and fibrosis. PLoS ONE 18:e0286257. 10.1371/journal.pone.0286257 37228085 10.1371/journal.pone.0286257 PMC10212180

[35] Reis-Costa A, Belew GD, Viegas I, Tavares LC, Meneses MJ, Patricio B, Gastaldelli A, Macedo MP, Jones JG (2024) The Effects of Long-Term High Fat and/or High Sugar Feeding on Sources of Postprandial Hepatic Glycogen and Triglyceride Synthesis in Mice. Nutrients 16(14):2186. 10.3390/nu16142186 39064628 10.3390/nu16142186 PMC11279633

[36] Moorthy M, Sundralingam U, Palanisamy UD (2021) Polyphenols as Prebiotics in the Management of High-Fat Diet-Induced Obesity: A Systematic Review of Animal Studies. Foods 10(2):299. 10.3390/foods10020299 33540692 10.3390/foods10020299 PMC7913110

[37] Tian Y, Ma J, Wang W, Zhang L, Xu J, Wang K, Li D (2016) Resveratrol supplement inhibited the NF-kappaB inflammation pathway through activating AMPKalpha-SIRT1 pathway in mice with fatty liver. Mol Cell Biochem 422:75–84. 10.1007/s11010-016-2807-x 27613163 10.1007/s11010-016-2807-x

[38] Zou Y, Li J, Lu C, Wang J, Ge J, Huang Y, Zhang L, Wang Y (2006) High-fat emulsion-induced rat model of nonalcoholic steatohepatitis. Life Sci 79:1100–1107. 10.1016/j.lfs.2006.03.021 16624332 10.1016/j.lfs.2006.03.021

[39] Al-Thepyani M, Algarni S, Gashlan H, Elzubier M, Baz L (2022) Evaluation of the Anti-Obesity Effect of Zeaxanthin and Exercise in HFD-Induced Obese Rats. Nutrients 14(23):4944. 10.3390/nu14234944 36500974 10.3390/nu14234944 PMC9737220

[40] Zhang XY, Guo CC, Yu YX, Xie L, Chang CQ (2020) Establishment of high-fat diet-induced obesity and insulin resistance model in rats. Beijing Da Xue Xue Bao Yi Xue Ban 52:557–563. 10.19723/j.issn.1671-167X.2020.03.024 32541992 10.19723/j.issn.1671-167X.2020.03.024 PMC7433434

[41] Zhang X, Huang C, Li X, Shangguan Z, Wei W, Liu S, Yang S, Liu Y (2020) HFD and HFD-provoked hepatic hypoxia act as reciprocal causation for NAFLD via HIF-independent signaling. BMC Gastroenterol 20:366. 10.1186/s12876-020-01515-5 33143650 10.1186/s12876-020-01515-5 PMC7640429

[42] Yaligar J, Gopalan V, Kiat OW, Sugii S, Shui G, Lam BD, Henry CJ, Wenk MR, Tai ES, Velan SS (2014) Evaluation of dietary effects on hepatic lipids in high fat and placebo diet fed rats by in vivo MRS and LC-MS techniques. PLoS ONE 9:e91436. 10.1371/journal.pone.0091436 24638096 10.1371/journal.pone.0091436 PMC3956606

[43] Xu J, Rong S, Gao H, Chen C, Yang W, Deng Q, Huang Q, Xiao L, Huang F (2017) A Combination of Flaxseed Oil and Astaxanthin Improves Hepatic Lipid Accumulation and Reduces Oxidative Stress in High Fat-Diet Fed Rats. Nutrients 9(3):271. 10.3390/nu9030271 28335388 10.3390/nu9030271 PMC5372934

[44] Giudetti AM, Vergara D, Longo S, Friuli M, Eramo B, Tacconi S, Fidaleo M, Dini L, Romano A, Gaetani S (2021) Oleoylethanolamide Reduces Hepatic Oxidative Stress and Endoplasmic Reticulum Stress in High-Fat Diet-Fed Rats. Antioxidants 10(8):1289. 10.3390/antiox10081289 34439537 10.3390/antiox10081289 PMC8389293

[45] Lasker S, Rahman MM, Parvez F, Zamila M, Miah P, Nahar K, Kabir F, Sharmin SB, Subhan N, Ahsan GU et al (2019) High-fat diet-induced metabolic syndrome and oxidative stress in obese rats are ameliorated by yogurt supplementation. Sci Rep 9:20026. 10.1038/s41598-019-56538-0 31882854 10.1038/s41598-019-56538-0 PMC6934669

[46] Alwahsh SM, Dwyer BJ, Forbes S, Thiel DH, Lewis PJ, Ramadori G (2017) Insulin Production and Resistance in Different Models of Diet-Induced Obesity and Metabolic Syndrome. Int J Mol Sci 18:285. 10.3390/ijms18020285 28134848 10.3390/ijms18020285 PMC5343821

[47] von Frankenberg AD, Marina A, Song X, Callahan HS, Kratz M, Utzschneider KM (2017) A high-fat, high-saturated fat diet decreases insulin sensitivity without changing intra-abdominal fat in weight-stable overweight and obese adults. Eur J Nutr 56:431–443 26615402 10.1007/s00394-015-1108-6 PMC5291812

[48] Krawczyk M, Burzynska-Pedziwiatr I, Wozniak LA, Bukowiecka-Matusiak M (2023) Impact of polyphenols on inflammatory and oxidative stress factors in diabetes mellitus: nutritional antioxidants and their application in improving antidiabetic therapy. Biomolecules. 10.3390/biom13091402 37759802 10.3390/biom13091402 PMC10526737

[49] Tabassum A, Mahboob T (2018) Role of peroxisome proliferator-activated receptor-gamma activation on visfatin, advanced glycation end products, and renal oxidative stress in obesity-induced type 2 diabetes mellitus. Hum Exp Toxicol 37:1187–1198. 10.1177/0960327118757588 29441829 10.1177/0960327118757588

[50] Portero-Otin M, de la Maza MP, Uribarri J (2023) Dietary Advanced Glycation End Products: Their Role in the Insulin Resistance of Aging. Cells 12(13):1684. 10.3390/cells12131684 37443718 10.3390/cells12131684 PMC10340703

[51] Detopoulou P, Voulgaridou G, Seva V, Kounetakis O, Desli I-I, Tsoumana D, Dedes V, Papachristou E, Papadopoulou S, Panoutsopoulos G (2024) Dietary Restriction of Advanced Glycation End-Products (AGEs) in Patients with Diabetes: A Systematic Review of Randomized Controlled Trials. Int J Mol Sci 25:11407. 10.3390/ijms252111407 39518960 10.3390/ijms252111407 PMC11546279

[52] Ziolkowska S, Binienda A, Jablkowski M, Szemraj J, Czarny P (2021) The Interplay between Insulin Resistance, Inflammation, Oxidative Stress, Base Excision Repair and Metabolic Syndrome in Nonalcoholic Fatty Liver Disease. Int J Mol Sci 22(20):11128. 10.3390/ijms222011128 34681787 10.3390/ijms222011128 PMC8537238

[53] Li J, Wang T, Liu P, Yang F, Wang X, Zheng W, Sun W (2021) Hesperetin ameliorates hepatic oxidative stress and inflammation via the PI3K/AKT-Nrf2-ARE pathway in oleic acid-induced HepG2 cells and a rat model of high-fat diet-induced NAFLD. Food Funct 12:3898–3918. 10.1039/D0FO02736G 33977953 10.1039/d0fo02736g

[54] Duan Y, Zeng L, Zheng C, Song B, Li F, Kong X, Xu K (2018) Inflammatory links between high fat diets and diseases. Front Immunol 9:2649. 10.3389/fimmu.2018.02649 30483273 10.3389/fimmu.2018.02649 PMC6243058

[55] Liu Y, Xu D, Yin C, Wang S, Wang M, Xiao Y (2018) IL-10/STAT3 is reduced in childhood obesity with hypertriglyceridemia and is related to triglyceride level in diet-induced obese rats. BMC Endocr Disord 18:39. 10.1186/s12902-018-0265-z 29895283 10.1186/s12902-018-0265-z PMC5998569

[56] Schaalan MF, Ramadan BK, Abd Elwahab AH (2018) Synergistic effect of carnosine on browning of adipose tissue in exercised obese rats; a focus on circulating irisin levels. J Cell Physiol 233:5044–5057. 10.1002/jcp.26370 29236301 10.1002/jcp.26370

[57] Han JM, Patterson SJ, Speck M, Ehses JA, Levings MK (2014) Insulin inhibits IL-10-mediated regulatory T cell function: implications for obesity. J Immunol 192:623–629. 10.4049/jimmunol.1302181 24323581 10.4049/jimmunol.1302181

[58] Wang X, Ba T, Cheng Y, Zhang P, Chang X (2021) Probiotics alleviate adipose inflammation in high-fat diet-induced obesity by restoring adipose invariant natural killer T cells. Nutrition 89:111285. 10.1016/j.nut.2021.111285 34116395 10.1016/j.nut.2021.111285

[59] Hamza RZ, Alsolami K (2023) Ameliorative effects of Orlistat and metformin either alone or in combination on liver functions, structure, immunoreactivity and antioxidant enzymes in experimentally induced obesity in male rats. Heliyon 9:e18724. 10.1016/j.heliyon.2023.e18724 37600390 10.1016/j.heliyon.2023.e18724 PMC10432992

[60] Echeverria F, Valenzuela R, Bustamante A, Alvarez D, Ortiz M, Soto-Alarcon SA, Munoz P, Corbari A, Videla LA (2018) Attenuation of High-Fat Diet-Induced Rat Liver Oxidative Stress and Steatosis by Combined Hydroxytyrosol- (HT-) Eicosapentaenoic Acid Supplementation Mainly Relies on HT. Oxid Med Cell Longev 2018:5109503. 10.1155/2018/5109503 30057681 10.1155/2018/5109503 PMC6051008

[61] Delli Bovi AP, Marciano F, Mandato C, Siano MA, Savoia M, Vajro P (2021) Oxidative Stress in Non-alcoholic Fatty Liver Disease. An Updated Mini Review Front Med (Lausanne) 8:595371. 10.3389/fmed.2021.595371 33718398 10.3389/fmed.2021.595371 PMC7952971

[62] Charradi K, Elkahoui S, Limam F, Aouani E (2013) High-fat diet induced an oxidative stress in white adipose tissue and disturbed plasma transition metals in rat: prevention by grape seed and skin extract. J Physiol Sci 63:445–455. 10.1007/s12576-013-0283-6 24158847 10.1007/s12576-013-0283-6 PMC11812467

[63] Alshabi AM, Shaikh IA (2022) Antidiabetic and antioxidant potential of *Gardenia latifolia* in type-2 diabetic rats fed with high-fat diet plus low-dose streptozotocin. Saudi Med J 43:881–890. 10.15537/smj.2022.43.8.20220258 35964948 10.15537/smj.2022.43.8.20220258 PMC9749673

[64] Pawar GR, Agrawal YO, Nakhate KT, Patil CR, Sharma C, Ojha S, Mahajan UB, Goyal SN (2022) Ghrelin alleviates depression-like behaviour in rats subjected to high-fat diet and diurnal rhythm disturbance. Am J Transl Res 14:7098–7108 36398212 PMC9641471

[65] Baz L, Algarni S, Al-Thepyani M, Aldairi A, Gashlan H (2022) Lycopene Improves Metabolic Disorders and Liver Injury Induced by a Hight-Fat Diet in Obese Rats. Molecules 27(22):7736. 10.3390/molecules27227736 36431836 10.3390/molecules27227736 PMC9699056

[66] Sztolsztener K, Dzieciol J, Chabowski A (2023) N-acetylcysteine acts as a potent anti-inflammatory agent altering the eicosanoid profile in the development of simple steatosis and its progression to hepatitis. Clin Exp Hepatol 9:386–395. 10.5114/ceh.2023.133106 38774197 10.5114/ceh.2023.133106 PMC11103808

[67] Jarukamjorn K, Jearapong N, Pimson C, Chatuphonprasert W (2016) A High-Fat, High-Fructose Diet Induces Antioxidant Imbalance and Increases the Risk and Progression of Nonalcoholic Fatty Liver Disease in Mice. Scientifica 2016:5029414. 10.1155/2016/5029414 27019761 10.1155/2016/5029414 PMC4785277

[68] Zhang N, Kong F, Jing X, Zhou J, Zhao L, Soliman MM, Zhang L, Zhou F (2022) Hongqu rice wines ameliorate high-fat/high-fructose diet-induced metabolic syndrome in rats. Alcohol Alcohol 57:776–787. 10.1093/alcalc/agac033 35922962 10.1093/alcalc/agac033

[69] Wang Y, Nakajima T, Gonzalez FJ, Tanaka N (2020) PPARs as Metabolic Regulators in the Liver: Lessons from Liver-Specific PPAR-Null Mice. Int J Mol Sci 21(6):2061. 10.3390/ijms21062061 32192216 10.3390/ijms21062061 PMC7139552

[70] Lin Y, Wang Y, Li PF (2022) PPARalpha: An emerging target of metabolic syndrome, neurodegenerative and cardiovascular diseases. Front Endocrinol 13:1074911. 10.3389/fendo.2022.1074911 10.3389/fendo.2022.1074911 PMC9800994 36589809

[71] Schlaepfer IR, Joshi M (2020) CPT1A-mediated Fat Oxidation, Mechanisms, and Therapeutic Potential. Endocrinology 161:bqz046. 10.1210/endocr/bqz046 31900483 10.1210/endocr/bqz046

[72] Zhao Y, Zhou Y, Wang D, Huang Z, Xiao X, Zheng Q, Li S, Long D, Feng L (2023) Mitochondrial Dysfunction in Metabolic Dysfunction Fatty Liver Disease (MAFLD). Int J Mol Sci 24(24):17514. 10.3390/ijms242417514 38139341 10.3390/ijms242417514 PMC10743953

[73] Hardwick JP, Osei-Hyiaman D, Wiland H, Abdelmegeed MA, Song BJ (2009) PPAR/RXR Regulation of Fatty Acid Metabolism and Fatty Acid omega-Hydroxylase (CYP4) Isozymes: Implications for Prevention of Lipotoxicity in Fatty Liver Disease. PPAR Res 2009:952734. 10.1155/2009/952734 20300478 10.1155/2009/952734 PMC2840373

[74] Abo-Zaid OA, Moawed FS, Ismail ES, Farrag MA (2023) beta-sitosterol attenuates high- fat diet-induced hepatic steatosis in rats by modulating lipid metabolism, inflammation and ER stress pathway. BMC Pharmacol Toxicol 24:31. 10.1186/s40360-023-00671-0 37173727 10.1186/s40360-023-00671-0 PMC10182633

[75] Zhang X, Li X, Fang H, Guo F, Li F, Chen A, Huang S (2019) Flavonoids as inducers of white adipose tissue browning and thermogenesis: signalling pathways and molecular triggers. Nutr Metab (Lond) 16:47. 10.1186/s12986-019-0370-7 31346342 10.1186/s12986-019-0370-7 PMC6637576

[76] Parlati L, Regnier M, Guillou H, Postic C (2021) New targets for NAFLD. JHEP Rep 3:100346. 10.1016/j.jhepr.2021.100346 34667947 10.1016/j.jhepr.2021.100346 PMC8507191

[77] Saponaro C, Gaggini M, Carli F, Gastaldelli A (2015) The Subtle Balance between Lipolysis and Lipogenesis: A Critical Point in Metabolic Homeostasis. Nutrients 7:9453–9474. 10.3390/nu7115475 26580649 10.3390/nu7115475 PMC4663603

[78] Wang X, Chen Y, Meng H, Meng F (2023) SREBPs as the potential target for solving the polypharmacy dilemma. Front Physiol 14:1272540. 10.3389/fphys.2023.1272540 38269061 10.3389/fphys.2023.1272540 PMC10806128

[79] Singh M, Thrimawithana T, Shukla R, Adhikari B (2020) Managing Obesity through Natural Polyphenols: A Review. Future Foods 1–2:100002. 10.1016/j.fufo.2020.100002

[80] Braczkowski MJ, Kufel KM, Kulinska J, Czyz DL, Dittmann A, Wiertelak M, Mlodzik MS, Braczkowski R, Soszynski D (2024) Pleiotropic Action of TGF-Beta in Physiological and Pathological Liver Conditions. Biomedicines 12(4):925. 10.3390/biomedicines12040925 38672279 10.3390/biomedicines12040925 PMC11048627

[81] Fabregat I, Caballero-Diaz D (2018) Transforming growth factor-beta-induced cell plasticity in liver fibrosis and hepatocarcinogenesis. Front Oncol 8:357. 10.3389/fonc.2018.00357 30250825 10.3389/fonc.2018.00357 PMC6139328

[82] Chen C-L, Lin Y-C (2022) Autophagy dysregulation in metabolic associated fatty liver disease: a new therapeutic target. Int J Mol Sci 23:10055. 10.3390/ijms231710055 36077452 10.3390/ijms231710055 PMC9456355

[83] Yang KL, Chang WT, Hong MY, Hung KC, Chuang CC (2017) Prevention of TGF-beta-induced early liver fibrosis by a maleic acid derivative anti-oxidant through suppression of ROS, inflammation and hepatic stellate cells activation. PLoS ONE 12:e0174008. 10.1371/journal.pone.0174008 28384213 10.1371/journal.pone.0174008 PMC5383026

