# Supplementary figures and images for: Effectiveness of dietary modifications in reversing damage induced by high-fat diet in rats
Source: J Physiol Biochem. 2026 Jul 14;82(1):67. doi: 10.1007/s13105-026-01205-y (PMC13369766; doi:10.1007/s13105-026-01205-y)

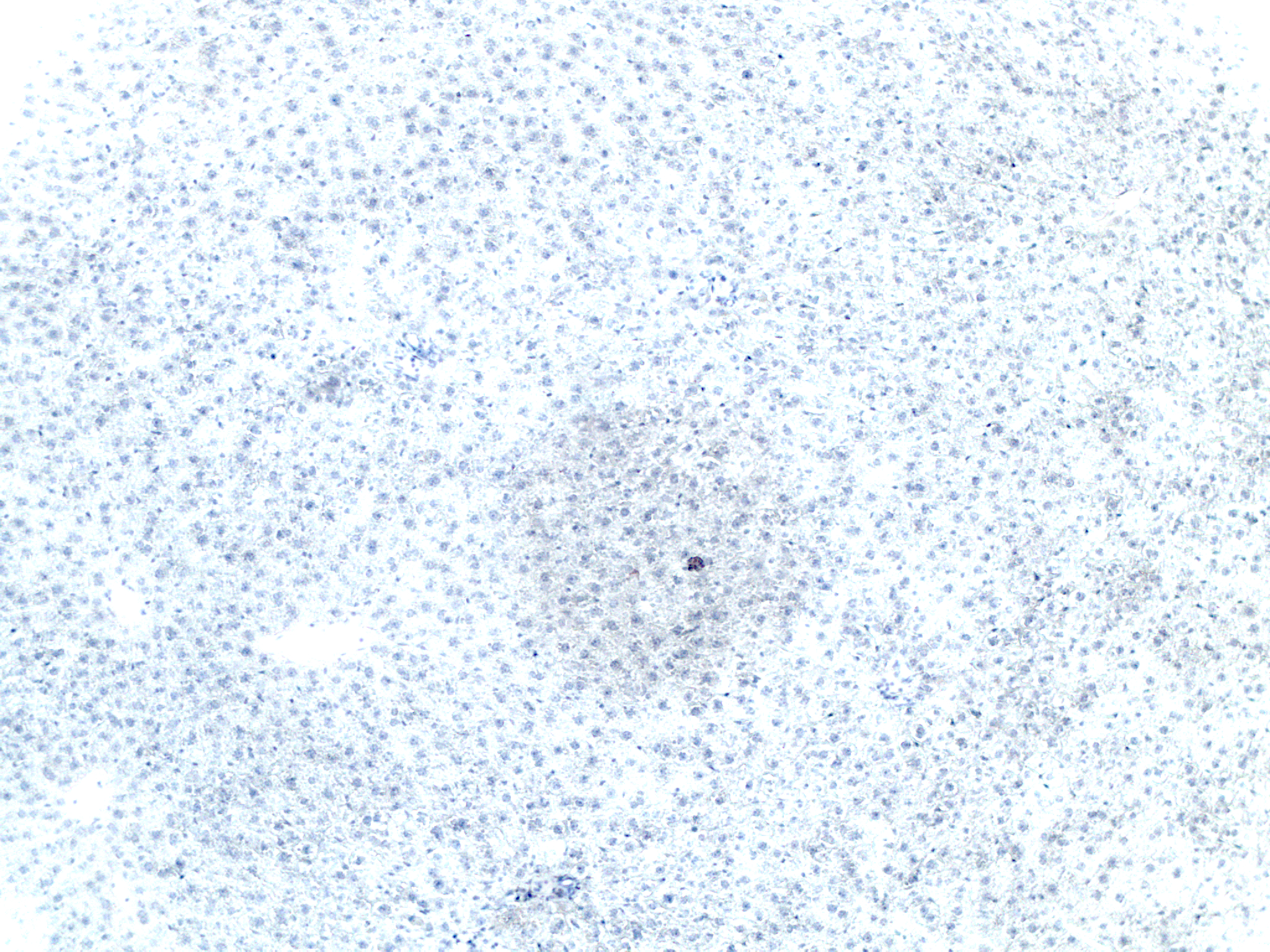

Supplement: Supplementary file 1 — Supplementary file1 (JPEG 5850 KB) [file 13105_2026_1205_MOESM1_ESM.jpeg]

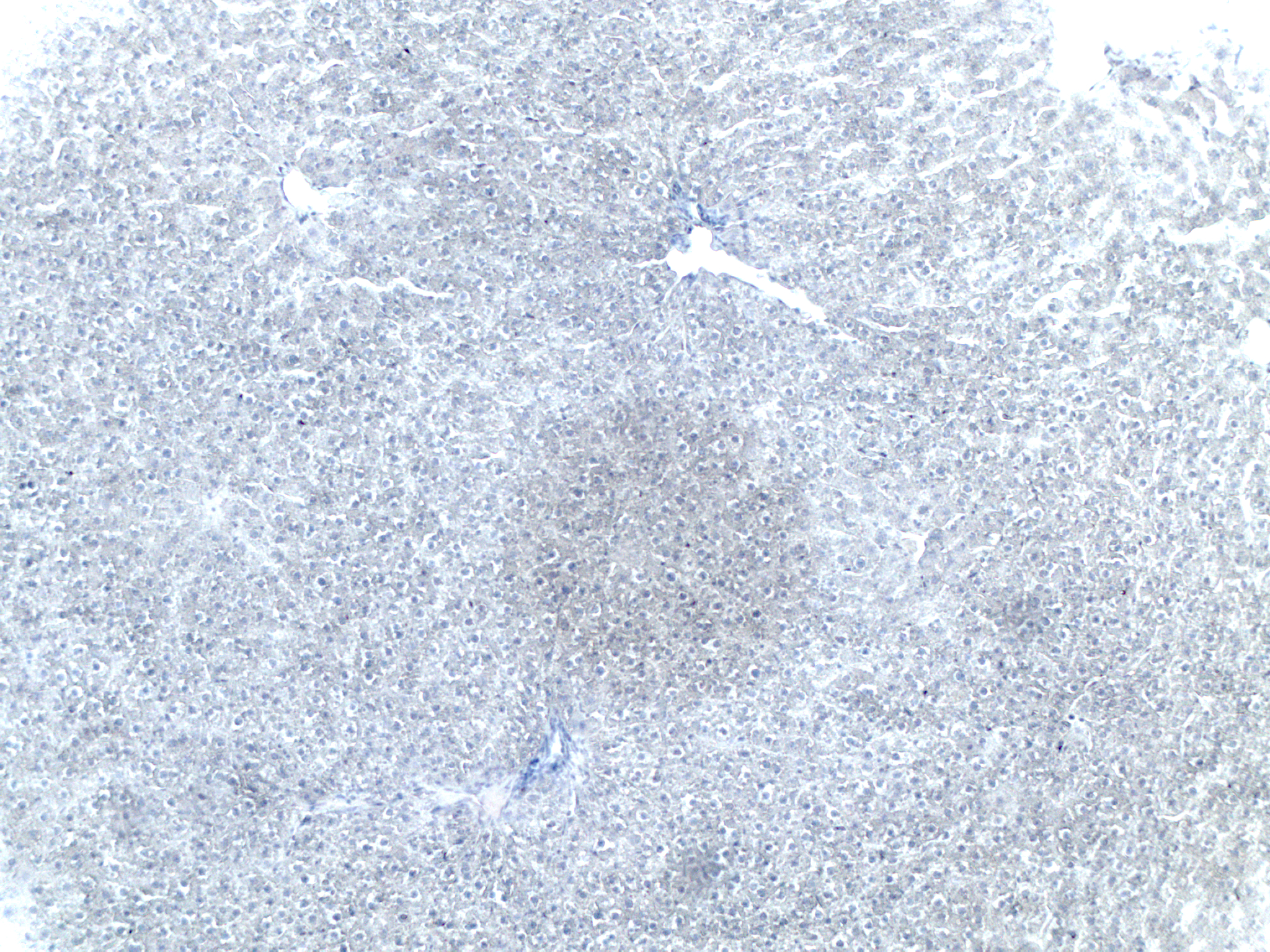

Supplement: Supplementary file 2 — Supplementary file2 (JPEG 6306 KB) [file 13105_2026_1205_MOESM2_ESM.jpeg]

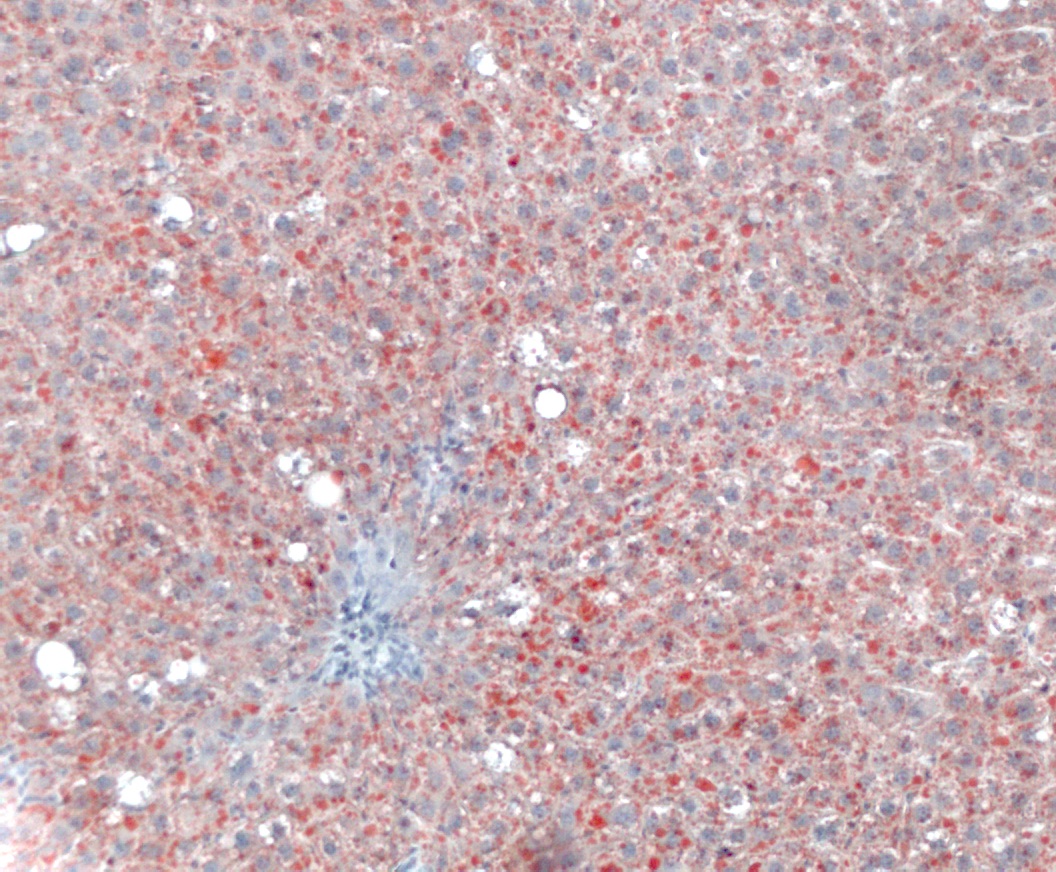

Supplement: Supplementary file 3 — Supplementary file3 (JPG 374 KB) [file 13105_2026_1205_MOESM3_ESM.jpg]

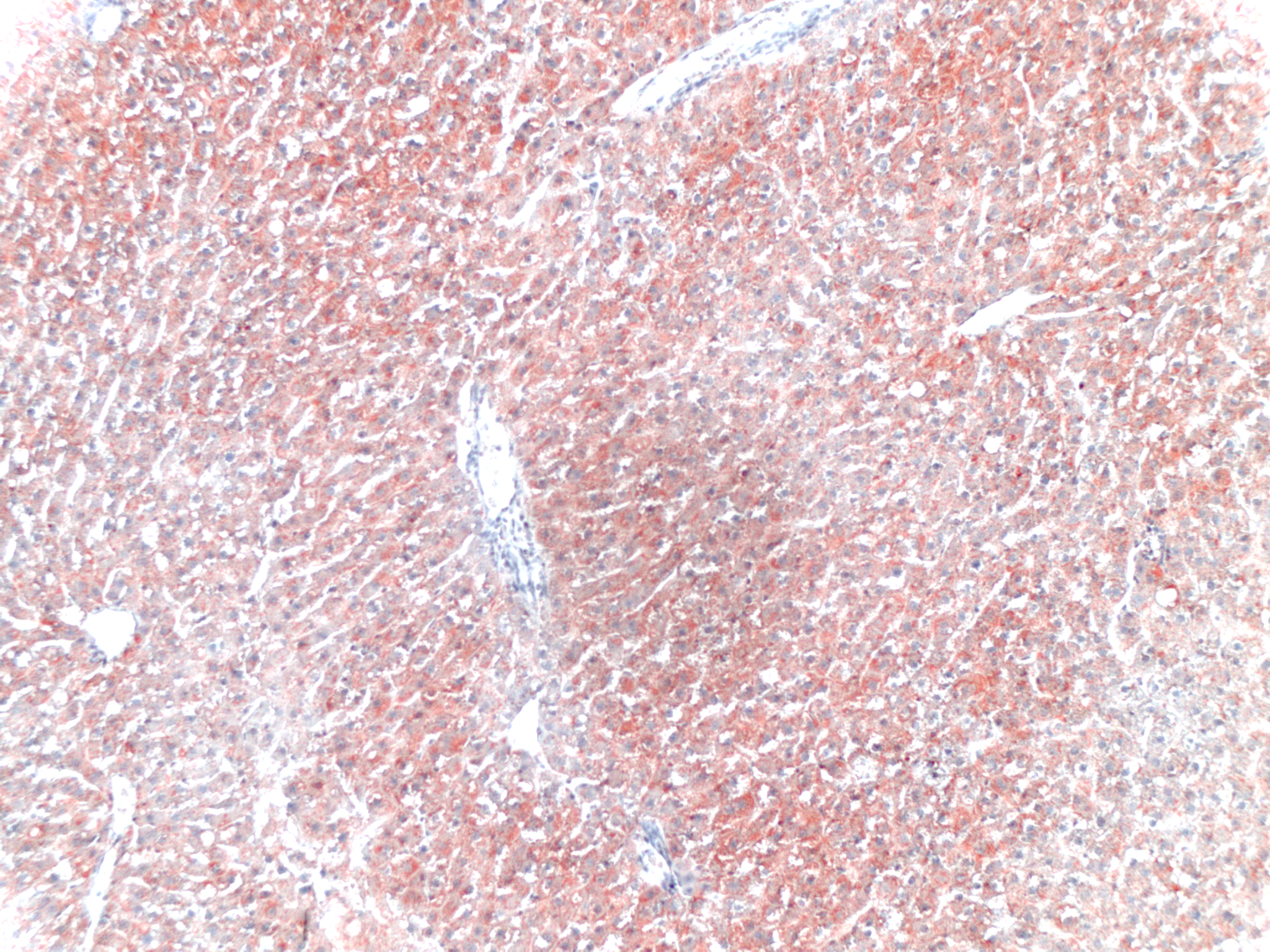

Supplement: Supplementary file 4 — Supplementary file4 (JPEG 5838 KB) [file 13105_2026_1205_MOESM4_ESM.jpeg]

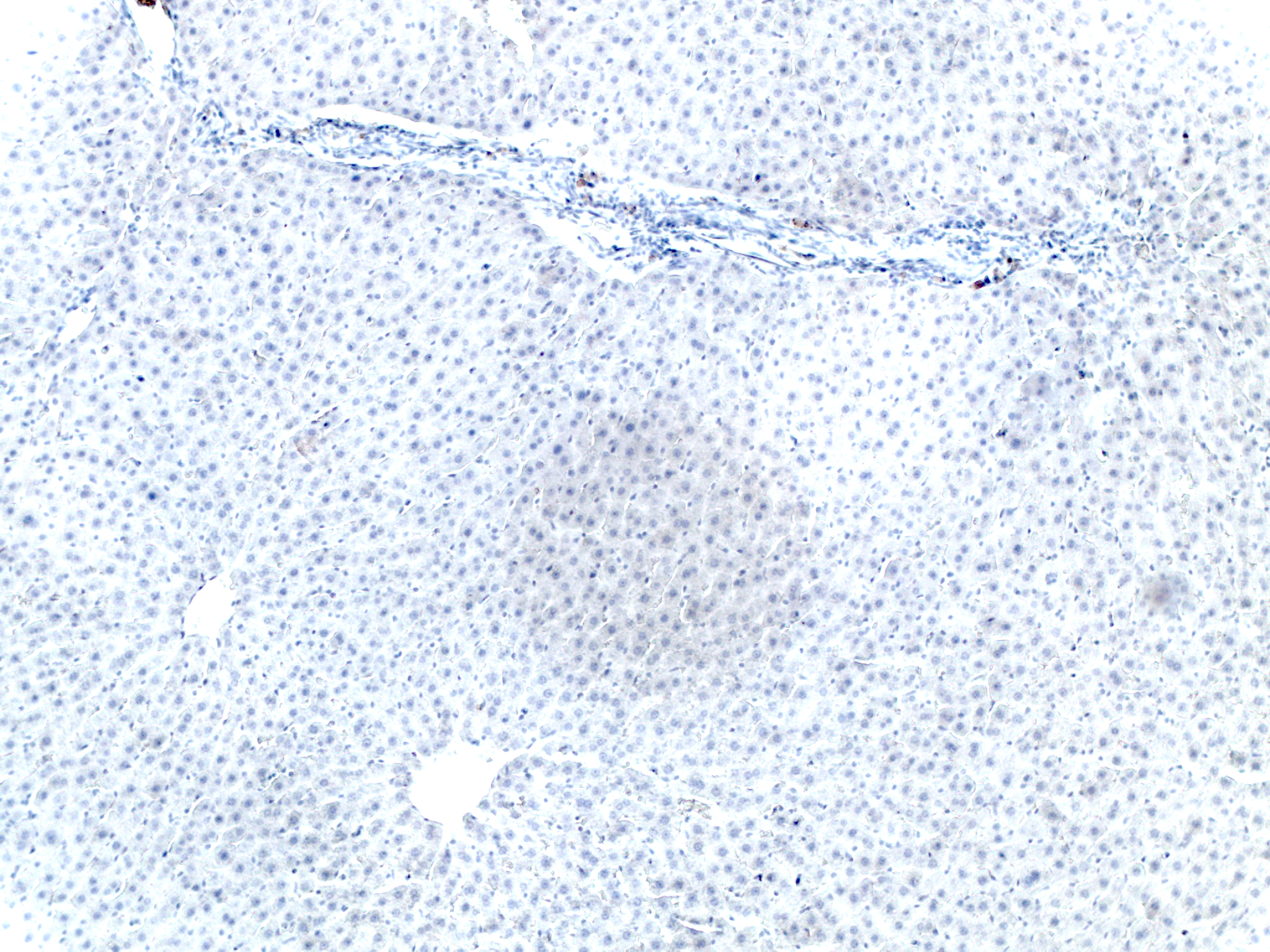

Supplement: Supplementary file 5 — Supplementary file5 (JPEG 5488 KB) [file 13105_2026_1205_MOESM5_ESM.jpeg]

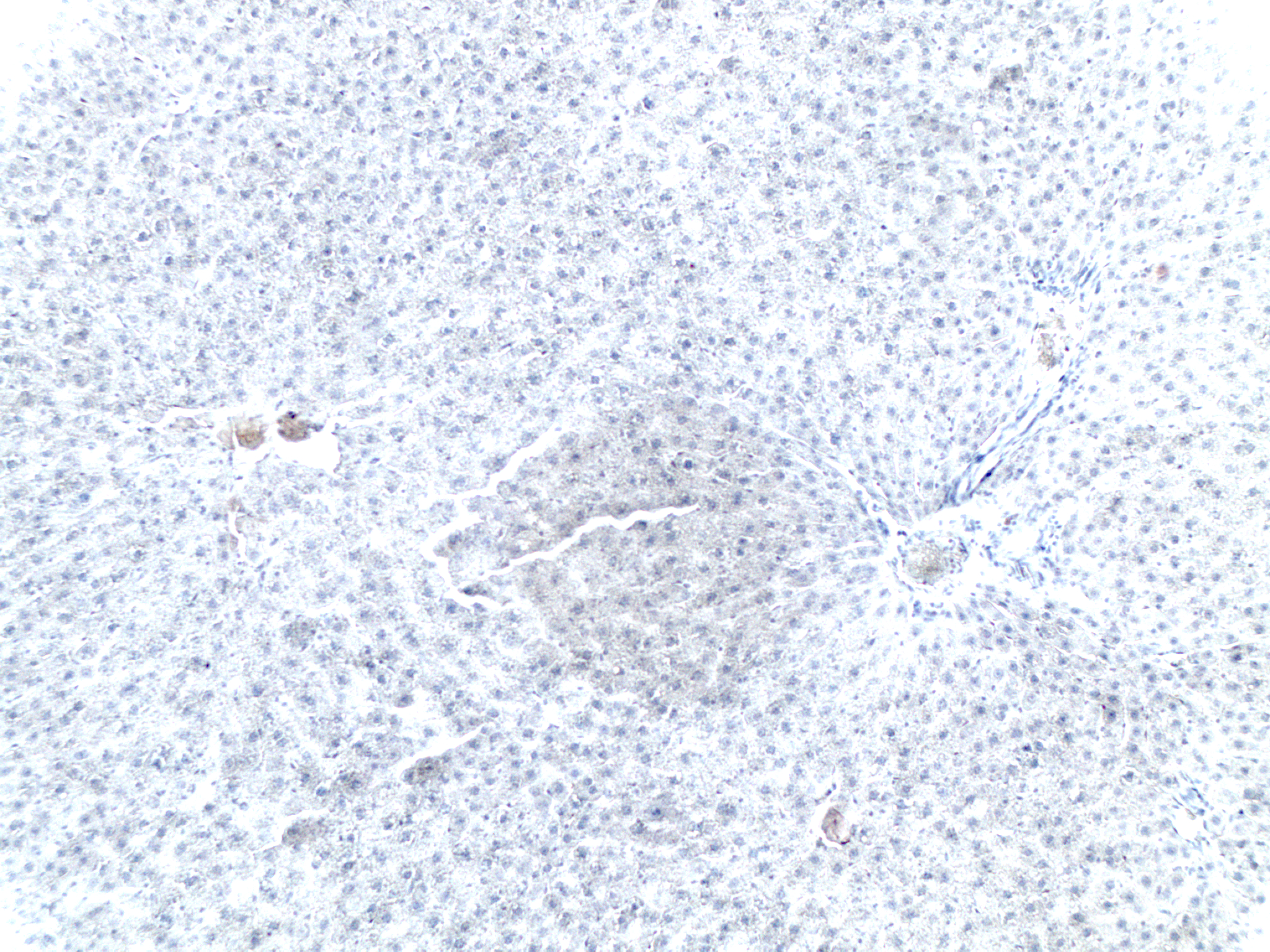

Supplement: Supplementary file 6 — Supplementary file6 (JPEG 5647 KB) [file 13105_2026_1205_MOESM6_ESM.jpeg]

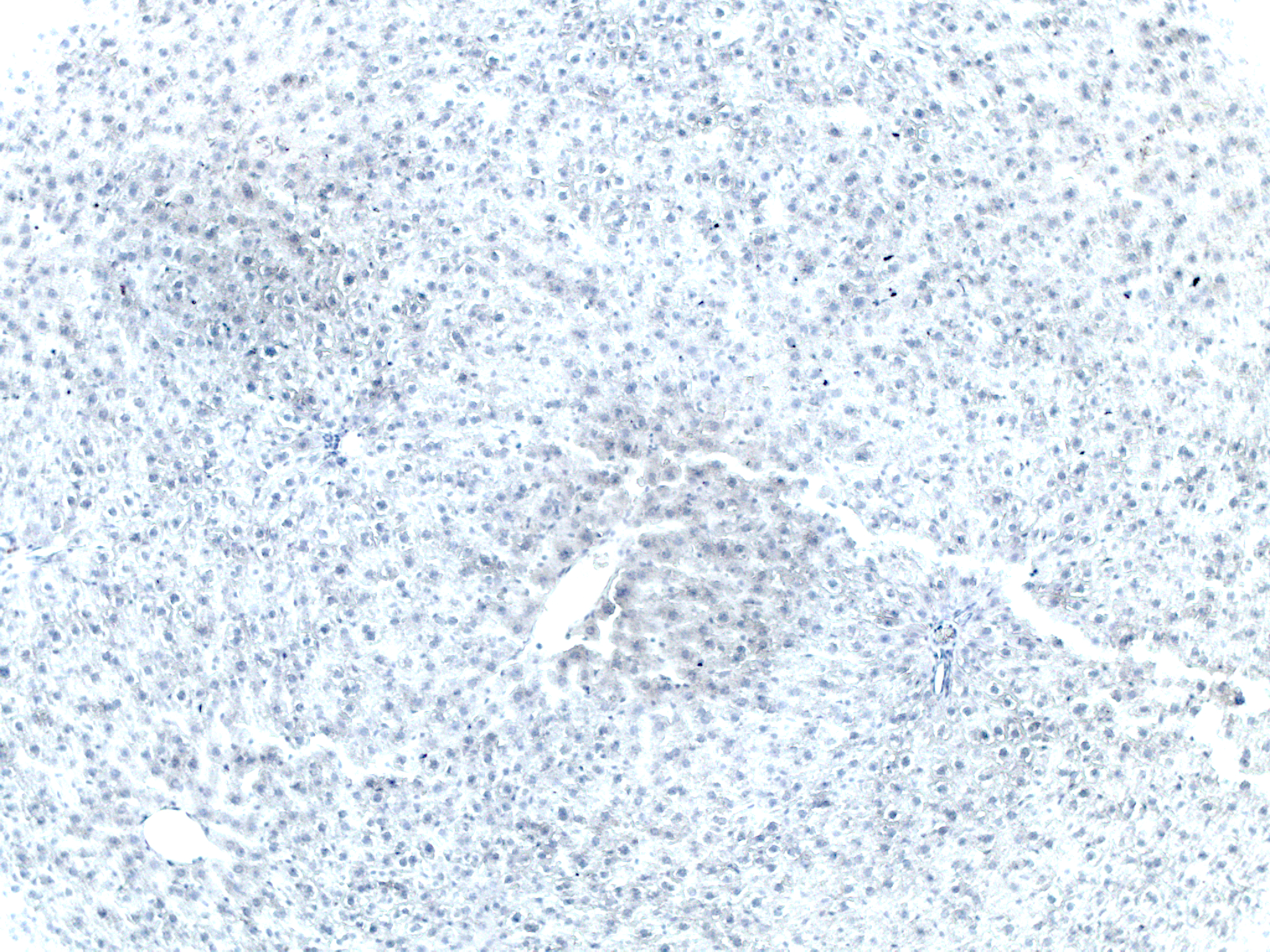

Supplement: Supplementary file 7 — Supplementary file7 (JPEG 6012 KB) [file 13105_2026_1205_MOESM7_ESM.jpeg]

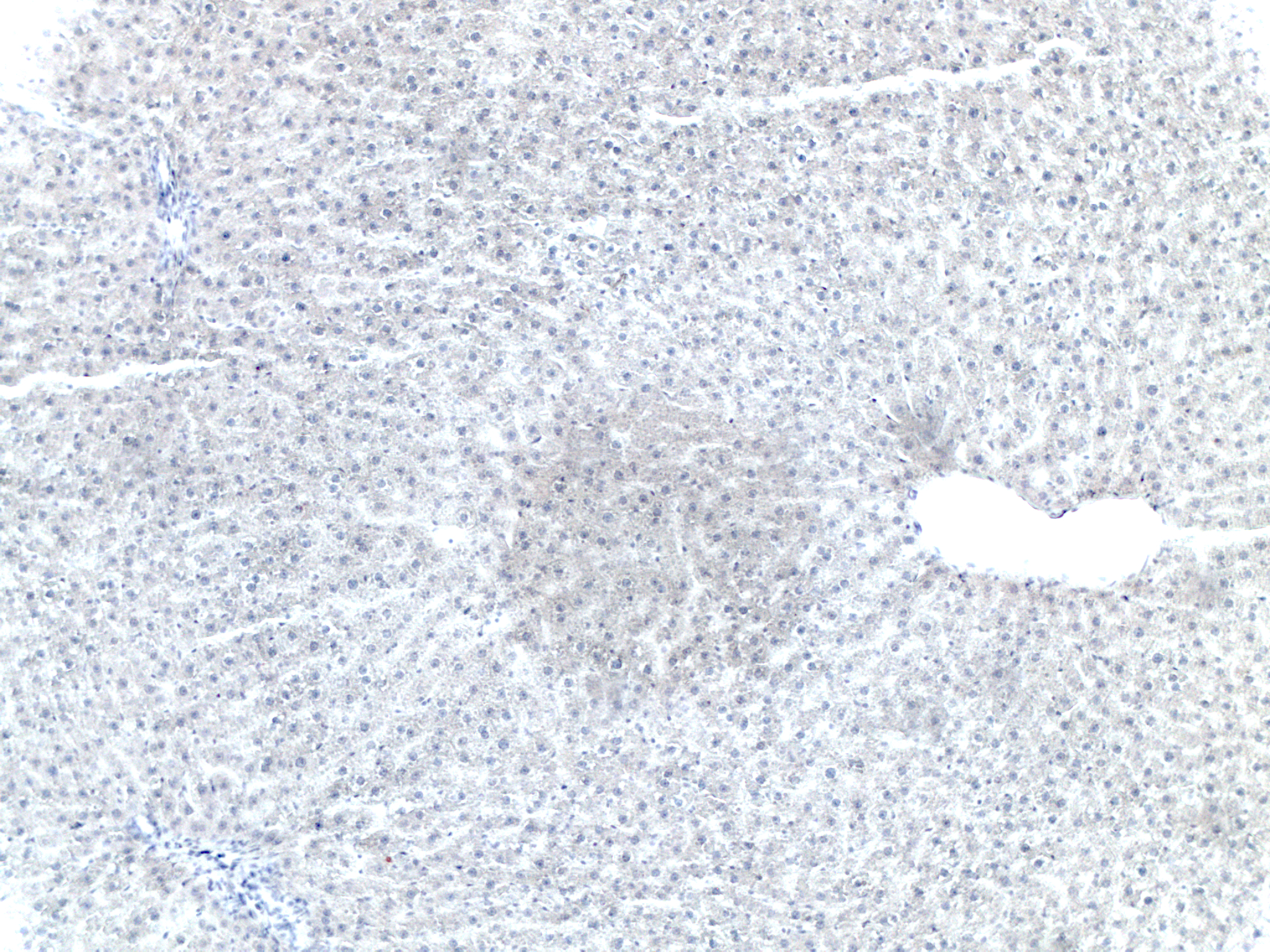

Supplement: Supplementary file 8 — Supplementary file8 (JPEG 6037 KB) [file 13105_2026_1205_MOESM8_ESM.jpeg]
