# Supplementary material for: Effectiveness of dietary modifications in reversing damage induced by high-fat diet in rats
Source: J Physiol Biochem. 2026 Jul 14;82(1):67. doi: 10.1007/s13105-026-01205-y (PMC13369766; doi:10.1007/s13105-026-01205-y)
Supplement: Supplementary file 9 — Supplementary file9 (DOCX 24 KB) [file 13105_2026_1205_MOESM9_ESM.docx]

**Supplementary Table S1.** Comparisons for the different variables in both female and male rats (n=7/sex and group) in plasma and liver from the control, high-fat diet (HFD), and HFD with change to standard (HFD+SD) or antioxidant-rich (HFD+Antiox) diets. Data are shown as F values, pairwise group comparisons, and 95% confidence intervals (CI). One-way ANOVA and LSD post hoc test.

| **Sample** | **Variable** | **Sex** | **F** | **Groups compared** | **95% CI** |
| --- | --- | --- | --- | --- | --- |
| - | Body weight | Females | F(3,27)=17.23 | Control *vs* HFD | [-122.6,-67.32] |
|  |  |  |  | HFD *vs* HFD + SD | [33.14,95.23] |
|  |  |  |  | HFD *vs* HFD + Antiox | [17.99,80.08] |
|  |  | Males | F(3,27)=17.58 | Control *vs* HFD | [-99.68,-53.97] |
|  |  |  |  | HFD *vs* HFD + SD | [20.51,68.41] |
|  |  |  |  | HFD *vs* HFD + Antiox | [30.01,77.91] |
| Plasma | Polyphenols content | Females | F(3,27)=13.29 | Control *vs* HFD + Antiox | [-3.390,-1.480] |
|  |  |  |  | HFD *vs* HFD + Antiox | [-3.351,-1.442] |
|  |  |  |  | HFD + SD *vs* HFD + Antiox | [-3.180,-1.270] |
|  |  | Males | F(3,27)=18.39 | Control *vs* HFD + Antiox | [-3.725,-1.865] |
|  |  |  |  | HFD *vs* HFD + Antiox | [-3.631,-1.772] |
|  |  |  |  | HFD + SD *vs* HFD + Antiox | [-3.526,-1.667] |
|  | Glucose | Females | F(3,27)=2.109 | Control *vs* HFD | [-54.60,-4.167] |
|  |  | Males | F(3,27)=2.011 | Control *vs* HFD | [-39.42,-3.185] |
|  | AGEs | Females | F(3,27)=20.22 | Control *vs* HFD | [-0.1794,-0.1006] |
|  |  |  |  | HFD *vs* HFD + SD | [0.03478,0.1136] |
|  |  |  |  | HFD *vs* HFD + Antiox | [0.06878,0.1476] |
|  |  | Males | F(3,27)=6.683 | Control *vs* HFD | [-0.3003,-0.1042] |
|  |  |  |  | HFD *vs* HFD + Antiox | [0.009994,0.2061] |
|  | IL-6 | Females | F(3,27)=5.013 | Control *vs* HFD | [-47.62,-10.16] |
|  |  |  |  | HFD *vs* HFD + SD | [9.024,46.49] |
|  |  |  |  | HFD *vs* HFD + Antiox | [9.873,47.34] |
|  |  | Males | F(3,27)=3.465 | Control *vs* HFD | [-98.83,-14.81] |
|  |  |  |  | HFD *vs* HFD + SD | [1.222,85.24] |
|  |  |  |  | HFD *vs* HFD + Antiox | [12.86,96.88] |
| Liver | Catalase | Females | F(3,27)=3.075 | Control *vs* HFD | [2.883,20.15] |
|  |  |  |  | HFD *vs* HFD + Antiox | [-18.69, -1.422] |
|  |  | Males | F(3,27)=2.462 | Control *vs* HFD | [1.054,10.47] |
|  |  |  |  | HFD *vs* HFD + SD | [-6.800,2.616] |
|  |  |  |  | HFD *vs* HFD + Antiox | [-8.875,0.5418] |
|  | Superoxide dismutase | Females | F(3,27)=3.197 | Control *vs* HFD | [0.02854,0.1585] |
|  |  | Males | F(3,27)=3.331 | Control vs HFD | [0.04634,0.2365] |
|  |  |  |  | HFD vs HFD + SD | [-0.1424,0.04774] |
|  | Glutathione peroxidase | Females | F(3,27)=16.45 | Control *vs* HFD | [3,378,6.739] |
|  |  |  |  | HFD *vs* HFD + Antiox | [-5.720,-2.360] |
|  |  | Males | F(3,27)=5.134 | Control *vs* HFD | [1.711,7.142] |
|  |  |  |  | HFD *vs* HFD + SD | [-6.127,0.6963] |
|  |  |  |  | HFD *vs* HFD + Antiox | [-7.077,-1.646] |
|  | Malondialdehyde | Females | F(3,27)=1.669 | Control *vs* HFD | [-0.5314,0.01257] |
|  |  | Males | F(3,27)=6.951 | Control *vs* HFD | [-0.7696,0.2510] |
|  |  |  |  | Control *vs* HFD + SD | [-0.6350,0.1164] |
|  |  |  |  | HFD *vs* HFD + Antiox | [0.1239,0.6425] |
|  | **Gene expression** |  |  |  |  |
| Liver | Il-6 | Females | F(3,27)=5.498 | Control *vs* HFD | [-81.62,-22.52] |
|  |  |  |  | HFD *vs* HFD + SD | [-4.200,54.90] |
|  |  | Males | F(3,27)=4.696 | Control *vs* HFD | [-56.88,-14.19] |
|  |  |  |  | HFD *vs* HFD + Antiox | [8.770,51.46] |
|  | IL-10 | Females | F(3,27)=5.695 | Control *vs* HFD | [10.34,54.56] |
|  |  |  |  | HFD *vs* HFD + Antiox | [-63.57,-19.36] |
|  |  | Males | F(3,27)=3.468 | Control *vs* HFD | [6.366,42.33] |
|  |  |  |  | HFD *vs* HFD + Antiox | [-41.78,-5.816] |
|  | CPT-1 | Females | F(3,27)=2.200 | HFD *vs* HFD + Antiox | [-143.4,-13.76] |
|  |  | Males | F(3,27)=4.579 | HFD *vs* HFD + Antiox | [-82.12,-22.75] |
|  | PPARα | Females | F(3,27)=4.261 | Control *vs* HFD | [5.936,50.90] |
|  |  |  |  | HFD *vs* HFD + SD | [-50.38,-5.419] |
|  |  |  |  | HFD *vs* HFD + Antiox | [-58.13,-13.17] |
|  | SREBP-1C | Females | F(3,27)=6.664 | Control *vs* HFD | [-120.7,-26.47] |
|  |  |  |  | HFD *vs* HFD + SD | [13.12,107.3] |
|  |  |  |  | HFD *vs* HFD + Antiox | [50.45,149.3] |
|  |  | Males | F(3,27)=14.96 | Control *vs* HFD | [-98.97,-47.00] |
|  |  |  |  | HFD *vs* HFD + SD | [31.56,83.54] |
|  |  |  |  | HFD *vs* HFD + Antiox | [43.93,95.90] |
|  | FASN | Females | F(3,27)=5.188 | Control *vs* HFD | [-67.49,-17.71] |
|  |  |  |  | HFD *vs* HFD + SD | [6.491,56.28] |
|  |  |  |  | HFD *vs* HFD + Antiox | [13.09,62.88] |
|  |  | Males | F(3,27)=2.520 | Control *vs* HFD | [-107.7,-6.693] |
|  |  |  |  | HFD *vs* HFD + Antiox | [6.776,107.8] |

**Abbreviations:** AGEs, advanced glycation end products; **CPT-1, carnitine palmitoyltransferase 1; FASN, fatty acid synthase;** IL-6, interleukin-6; **Il-10, interleukin-10; PPARα, peroxisome proliferator-activated receptor alpha; SREBP-1C, sterol regulatory element-binding protein 1C.**
